# Design of Fixed-Time Fault-Disturbance Estimator for Emergency Rescue Quadrotor UAV Under Multi-Stage Mission Conditions

**DOI:** 10.3390/s26155004

**Published:** 2026-08-06

**Authors:** Lihong Rong, Chengguo Han, Fuzhu Ding, Tianshuo Li, Siwen Chen, Zhimin Tong

**Affiliations:** College of Mechanical and Electrical Engineering, Qingdao Agricultural University, Qingdao 266109, China; ronglh@qau.edu.cn (L.R.); 20252111026@stu.qau.edu.cn (S.C.)

**Keywords:** quadrotor UAV, emergency rescue, fault-disturbance estimation, fixed-time estimator, lumped fault disturbance

## Abstract

In this paper, the fixed-time fault-disturbance estimation problem for an emergency rescue quadrotor UAV during the takeoff–mission execution–landing process is investigated. Considering the complex operating conditions of rescue missions, actuator efficiency loss, actuator bias faults, rescue-environment airflow disturbances, payload-induced disturbances, near-ground aerodynamic effects, and unmodeled dynamic uncertainties are uniformly represented as a lumped fault disturbance entering the angular-velocity dynamic channel. A multi-stage disturbance model is first established according to the takeoff stage, mission execution stage, and landing stage. Then, a fixed-time fault-disturbance estimator with stage-dependent gains is designed to estimate the lumped disturbance. Based on matrix Lyapunov functions and fixed-time stability theory, it is proven that the angular-velocity estimation error and the lumped fault-disturbance estimation error can enter and remain in a small bounded neighborhood within a fixed time independent of the initial conditions. Moreover, the bounded disturbance jumps at the stage-transition instants are analyzed, and the post-transition recovery property of the estimator is established. The simulation results under a practical emergency rescue scenario show that the proposed estimator can reconstruct the main variation trends of the roll, pitch, and yaw channel lumped disturbances, keep the estimation error bounded, and recover after stage-transition-induced disturbance variations.

## 1. Introduction

In recent years, unmanned aerial vehicles (UAVs) have been widely used in disaster monitoring, emergency rescue, search and rescue, communication relay, and material delivery missions. Among the different UAV platforms, quadrotor UAVs have attracted extensive attention because of their vertical takeoff and landing capability, hovering ability, compact structure, and high maneuverability. These characteristics make quadrotor UAVs suitable for emergency rescue missions in mountainous areas, urban ruins, flooded regions, and other complex environments where ground rescue equipment may have difficulty entering. Lyu et al. reviewed the development of UAV-based search and rescue systems and summarized the roles of UAVs in target perception, localization, and rescue decision support [1]. Sanz-Martos et al. systematically analyzed drone applications in emergency and urgent care, showing that drones can reduce response time and improve emergency medical service efficiency [2]. In parallel with these application-oriented studies, theoretical research on filtering and reliable control has provided relevant foundations for systems subject to mode transitions, time delays, and actuator faults. Wei et al. investigated the $H_{\infty }$ memory filtering problem for semi-Markovian jump time-delay systems by simultaneously considering the mode-transition mechanism and time-delay effects [3]. Qiu et al. developed a reliable control approach for piecewise affine systems with actuator faults, providing a useful theoretical basis for the fault-tolerant analysis of systems with mode-dependent dynamics [4]. At the application level, Oh and Han proposed an autonomous UAV smart search system for disaster rescue, which emphasized autonomous search and tracking of people in distress when communication with ground control stations is limited [5]. Cao et al. studied multi-UAV task allocation and path planning in emergency rescue scenarios with uncertain requirements, providing a reference for cooperative rescue mission planning [6]. Collectively, these studies provide important foundations for emergency UAV applications, filtering under mode transitions, and reliable control under actuator faults. However, the fixed-time estimation of stage-dependent lumped fault disturbances during the complete rescue flight process of a quadrotor UAV has not been sufficiently investigated.

For quadrotor UAVs operating in emergency rescue environments, external disturbances, model uncertainties, and actuator faults may occur simultaneously. During takeoff, the vehicle may be affected by near-ground aerodynamic effects and actuator output inconsistency. During mission execution, it may suffer from wind gusts, thermal airflow, payload variation, and unmodeled dynamic uncertainties. During landing, the near-ground aerodynamic effect and attitude adjustment uncertainty may become significant again. Therefore, the disturbance acting on the quadrotor is generally time-varying and stage-dependent. To improve the robustness of quadrotor systems under external disturbances, Ai and Yu proposed a fixed-time trajectory tracking method for a quadrotor with external disturbances by combining differential flatness and sliding-mode control [7]. From the perspective of fault estimation under stochastic mode transitions, Rong et al. developed an extended-state-based fault-estimation method for semi-Markov jump neural networks, demonstrating the applicability of the extended state method to fault reconstruction in systems with mode-dependent dynamics [8]. Lin et al. developed a decoupling control method for quadrotor UAVs using dynamic surface control and a sliding-mode disturbance observer, which improved the anti-disturbance capability of the system [9]. Gong et al. investigated fixed-time integral-type sliding-mode attitude stabilization for a quadrotor UAV under actuator failures, demonstrating the importance of fixed-time response in faulty quadrotor systems [10]. Labbadi and Cherkaoui designed a robust adaptive backstepping fast terminal sliding-mode controller for uncertain quadrotor UAVs, which enhanced the tracking performance under uncertainties [11]. Ahmed et al. studied disturbance-observer-based tracking control for quadrotors with high-order disturbances, providing an effective way to estimate and compensate complex external disturbances [12]. Zhao et al. proposed a high-order sliding-mode observer-based trajectory tracking method for a quadrotor UAV with uncertain dynamics, showing that observer-based compensation can improve tracking robustness [13]. Although these methods improve the disturbance rejection capability of quadrotor UAVs, most of them are designed for continuous flight conditions and do not explicitly consider the disturbance jumps caused by mission-stage transitions.

Actuator faults are another important factor affecting the safety of quadrotor UAVs. In practical rescue missions, rotor efficiency loss, actuator bias, and partial actuator faults may degrade attitude stability and even lead to mission failure. Tang et al. proposed an observer-based finite-time fault-tolerant attitude control method for a quadrotor with actuator faults, where finite-time estimation and compensation were used to improve fault tolerance [14]. Wang et al. studied active fault-tolerant control for a quadrotor helicopter against actuator faults and model uncertainties, showing that active fault compensation can improve system safety under fault conditions [15]. Hou et al. investigated nonsingular terminal sliding-mode control for a quadrotor UAV with a total rotor failure, providing a control strategy for severe actuator fault cases [16]. Mallavalli and Fekih developed a fault-tolerant tracking control method for a quadrotor UAV subject to simultaneous actuator faults and exogenous disturbances, indicating that faults and disturbances should be considered together [17]. Yang et al. proposed a sliding-mode fault-tolerant control method for a quadrotor with varying load and actuator fault, which is closely related to rescue missions involving payload changes [18]. Liu et al. designed an anti-saturation adaptive finite-time neural-network-based fault-tolerant tracking controller for a quadrotor UAV with external disturbances, addressing the effects of actuator saturation and uncertainty [19]. Tripathi et al. proposed a disturbance-observer-based intelligent finite-time sliding-mode flight controller for an autonomous quadrotor, further demonstrating the effectiveness of combining disturbance observation with finite-time control [20]. These studies provide useful methods for quadrotor fault-tolerant control, but their main focus is closed-loop control design rather than the independent fixed-time estimation of stage-dependent lumped fault disturbances.

In addition to conventional finite-time and sliding-mode methods, disturbance-observer-based robust fault-tolerant control has also been widely studied for quadrotor UAVs. Liu et al. proposed a fixed-time disturbance-observer-based robust fault-tolerant tracking control method for a quadrotor UAV subject to input delay, where the fixed-time disturbance observer was used to improve compensation performance [21]. Wang et al. designed a disturbance-observer-based nonsingular fast terminal sliding-mode fault-tolerant controller for a quadrotor UAV with external disturbances and actuator faults, showing that observer-based compensation can enhance robustness under compound faults [22]. Ma et al. introduced a nonlinear extended-state observer into prescribed performance control for quadrotor UAVs with attitude and input saturation constraints, which improved the estimation of total disturbances [23]. Gong et al. proposed a prescribed-time extended-state observer and prescribed performance control method for quadrotor UAVs against actuator faults, highlighting the importance of preassigned convergence performance in fault-tolerant control [24]. Hu et al. developed a disturbance-observer-enhanced adaptive fault-tolerant control method for a quadrotor UAV against actuator faults and disturbances, where the disturbance observer was used to support adaptive compensation [25]. Miranda-Moya et al. designed a fixed-time extended-observer-based adaptive sliding-mode controller for quadrotor UAVs subject to severe turbulent wind, showing that fixed-time observer design is useful for rapid disturbance estimation in complex wind environments [26]. Wang et al. proposed an adaptive sliding-mode fault-tolerant control method for a quadrotor UAV with actuator faults and model uncertainties, demonstrating the importance of robustness against both faults and uncertainties [27]. These works have significantly improved the fault-tolerant control performance of quadrotor UAVs. Nevertheless, most of them mainly use disturbance estimation as an auxiliary part of controller design, and the stage-dependent evolution of lumped fault disturbances during a complete takeoff–mission execution–landing rescue process is still not fully considered.

Beyond observer-assisted fault-tolerant control, fixed-time, prescribed-time and finite-time convergence techniques have recently attracted increasing attention in quadrotor UAV control because they can provide explicit convergence-time performance and improved transient response. Jeong et al. proposed a variable disturbance-observer-based control strategy for quadrotor UAVs, indicating that disturbance observers are effective tools for handling time-varying disturbances [28]. Mai et al. developed a nonlinear extended-state observer and prescribed performance fault-tolerant control method for quadrotor UAVs against compound faults, which is relevant to the estimation and compensation of multiple unknown effects [29]. Cai et al. investigated fixed-time trajectory tracking control of a quadrotor UAV under time-varying wind disturbances and provided experimental validation, showing the practical value of fixed-time techniques for quadrotor systems [30]. Xu et al. proposed a fixed-time disturbance-observer-based model predictive robust trajectory tracking control method for quadrotors in which the fixed-time observer estimated lumped fault disturbances for robust tracking [31]. Song et al. studied safety-critical fixed-time formation control of quadrotor UAVs with disturbances and obstacle collision risk, extending fixed-time methods to multi-quadrotor safety-critical missions [32]. Liu et al. proposed a robust fault-tolerant control method for quadrotor UAVs with parameter uncertainties and actuator faults, further emphasizing the need to handle actuator faults and uncertainties simultaneously [33]. Kuang et al. designed an adaptive sliding-mode control method for trajectory tracking of uncertain quadrotor UAVs, which improved tracking robustness under uncertain dynamics [34]. Tran et al. developed an integrated robust tracking framework combining detailed quadrotor modeling, sliding-mode control with radial-basis-function neural network approximation, particle-swarm-based parameter optimization, and trajectory planning [35]. Ranjan and Majhi proposed a fixed-time state-observer-based robust adaptive neural fault-tolerant control method for a quadrotor UAV, where fixed-time state observation was combined with adaptive neural compensation [36]. Zhang et al. investigated finite-time fault-tolerant tracking control for a quadrotor UAV with unknown mixed faults and external disturbances, providing a reference for handling mixed fault-disturbance conditions [37]. Yan et al. proposed an adaptive finite-time fault-tolerant control scheme for UAV systems under simultaneous actuator faults and external disturbances, which further shows the importance of finite-time response under compound faults [38]. Although these recent studies have advanced fixed-time and finite-time control of quadrotor UAVs, the existing literature still mainly focuses on trajectory tracking, attitude control, or formation control. The fixed-time estimation of multi-stage lumped fault disturbances, especially under bounded disturbance jumps at mission-stage transitions, remains insufficiently investigated.

Based on the above analysis, it can be seen that the existing studies have provided important foundations for emergency UAV applications, quadrotor robust control, fault-tolerant control, disturbance observation, and fixed-time convergence analysis. However, several issues still need further investigation. First, most emergency rescue UAV studies focus on search, perception, path planning, or task allocation, while the flight safety problem caused by actuator faults and disturbances in quadrotor dynamics is not deeply addressed. Second, many quadrotor robust control and fault-tolerant control methods consider external disturbances or actuator faults, but they usually model them under a continuous flight condition rather than a complete takeoff–mission execution–landing process. Third, the existing fixed-time observers are often embedded in control systems, and the independent fixed-time estimation problem of stage-dependent lumped fault disturbances has not been sufficiently discussed. Fourth, when a quadrotor UAV switches between different rescue mission stages, the lumped fault disturbance may exhibit bounded jumps, and the post-transition recovery property of the estimation error requires further analysis.

Motivated by these observations, this paper investigates the fixed-time lumped fault-disturbance estimation problem for an emergency rescue quadrotor UAV during the takeoff–mission execution–landing process. Different from studies that only consider single-stage disturbances or directly design fault-tolerant controllers, this paper constructs a multi-stage lumped fault-disturbance model for the angular-velocity dynamic channel. The effects of actuator efficiency loss, actuator bias faults, rescue-environment airflow disturbances, payload-induced disturbances, near-ground aerodynamic effects, and unmodeled dynamic uncertainties are uniformly represented as a lumped fault disturbance Δ(t). Then, a fixed-time fault-disturbance estimator with stage-dependent gains is designed to estimate the lumped fault disturbance. The estimation error system is analyzed by matrix Lyapunov functions, and the bounded disturbance jumps at the stage-transition instants are also considered.

The main contribution of this paper lies in developing a unified fixed-time estimation framework for stage-dependent lumped fault disturbances in emergency rescue quadrotor UAV missions. Unlike conventional studies that usually focus on single-stage flight conditions or regard disturbance estimation only as an auxiliary part of controller design, this paper considers the complete takeoff–mission execution–landing process and constructs a multi-stage lumped fault-disturbance model for the angular-velocity dynamic channel. Within this framework, actuator-fault effects, payload-induced disturbances, rescue-environment airflow disturbances, near-ground aerodynamic effects, and unmodeled dynamic uncertainties are uniformly incorporated into a single disturbance representation. Based on this model, a fixed-time fault-disturbance estimator with stage-dependent gains is designed to achieve rapid estimation under different mission stages. Furthermore, by employing matrix Lyapunov analysis and fixed-time stability theory, it is shown that the angular-velocity estimation error and the lumped fault-disturbance estimation error can enter and remain in a small bounded neighborhood within a fixed time independent of the initial conditions. The bounded disturbance jumps caused by stage transitions are also considered, and the post-transition recovery property of the estimator is established.

## 2. Problem Formulation and Multi-Stage Lumped Fault Disturbance Modeling

In this section, a quadrotor UAV attitude dynamics model with fault effects is established, and a multi-stage lumped fault-disturbance model is formulated for the takeoff–mission execution–landing process of emergency rescue missions. Based on this model, the multi-stage lumped fault-disturbance estimation problem is subsequently formulated.

### 2.1. Quadrotor UAV Attitude Modeling and Fault Representation

The attitude-tracking flight dynamic model considered in this paper is given as follows [39]:

(1) $$\left\{\begin{array}{cc} \dot{\phi } & =p+\tan\theta \left(q\sin\phi +r\cos\phi \right)\\ \dot{\theta } & =q\cos\phi -r\sin\phi \\ \dot{\psi } & =\frac{q\sin\phi +r\cos\phi }{\cos\theta } \end{array}\right.$$

(2) $$\left\{\begin{array}{cc} \dot{p} & =\frac{J_{y}-J_{z}}{J_{x}}qr+\frac{1}{J_{x}}\tau_{\phi }\\ \dot{q} & =\frac{J_{z}-J_{x}}{J_{y}}pr+\frac{1}{J_{y}}\tau_{\theta }\\ \dot{r} & =\frac{J_{x}-J_{y}}{J_{z}}pq+\frac{1}{J_{z}}\tau_{\psi } \end{array}\right.$$

where $J_{x}$, $J_{y}$, and $J_{z}$ denote the moments of inertia about the roll, pitch, and yaw axes, respectively. The control moments $\tau_{\phi }$, $\tau_{\theta }$, and $\tau_{\psi }$ are generated by the differential thrusts of the four rotors. Define the attitude-angle vector, the body-fixed angular-velocity vector, and the control-input vector as $x_{1}={\left[\phi ,\theta ,\psi \right]}^{T}$, $x_{2}={\left[p,q,r\right]}^{T}$, and $u={\left[\tau_{\phi },\tau_{\theta },\tau_{\psi }\right]}^{T}$, respectively. Based on (1), the complete attitude kinematic relationship can be rewritten as

$${\dot{x}}_{1}=W\left(x_{1}\right)x_{2}$$

where

$$W\left(x_{1}\right)=\left[\begin{array}{ccc} 1 & \sin\phi \tan\theta & \cos\phi \tan\theta \\ 0 & \cos\phi & -\sin\phi \\ 0 & \frac{\sin\phi }{\cos\theta } & \frac{\cos\phi }{\cos\theta } \end{array}\right]$$

The mapping matrix $W(x_{1})$ is well defined for cosθ≠0, excluding only the Euler-angle singularity at θ=±π/2. No small-angle approximation is imposed; hence, the complete nonlinear coupling between the Euler-angle rates and the body-fixed angular velocities is retained. The importance of preserving the complete nonlinear attitude kinematics in constrained attitude-reorientation problems has also been highlighted in recent studies [40]. Accordingly, the nominal attitude dynamics can be expressed in the following affine nonlinear form:

(3) $$\left\{\begin{array}{c} {\dot{x}}_{1}=W\left(x_{1}\right)x_{2}\\ {\dot{x}}_{2}=f\left(x\right)+g\left(x\right)u \end{array}\right.$$

where f(x) collects the inertial coupling terms in the angular-velocity dynamics, while g(x) denotes the input gain matrix from the control input to the angular-velocity dynamics. In this study, the proposed estimator is designed for the lumped fault disturbance entering the angular-velocity dynamic channel. Therefore, the subsequent estimator design and convergence analysis are based on the second subsystem of (3). During the takeoff–mission execution–landing process, faults may occasionally occur in the UAV system. It is assumed that $u_{\mathrm{fault}}\left(t\right)$ denotes the equivalent actuator fault input induced by rotor efficiency loss, motor-thrust bias, and thrust-allocation errors, and $d_{\mathrm{fault}}\left(t\right)$ represents the unknown fault entering the angular-velocity dynamic channel. Then, based on (3), the UAV model with faults is given as follows:

(4) $$\left\{\begin{array}{cc} {\dot{x}}_{1} & =W\left(x_{1}\right)x_{2}\\ {\dot{x}}_{2} & =f\left(x\right)+g\left(x\right)\left(u+u_{\mathrm{fault}}\left(t\right)\right)+d_{\mathrm{fault}}\left(t\right) \end{array}\right.$$

### 2.2. Multi-Stage Lumped Fault-Disturbance Modeling for Emergency Rescue Missions

During emergency rescue missions, quadrotor UAVs usually operate under time-critical and uncertain conditions. Different from routine flight tasks, the UAV may be required to take off from a temporary site, execute low-altitude rescue missions in a disturbed environment, and land on a nonstandard recovery area. Therefore, the disturbance mechanism varies significantly among the takeoff, mission execution, and landing stages. To describe the multi-stage characteristic, a mission-stage variable is introduced as:

(5) $$\sigma \left(t\right)=\left\{\begin{array}{cc} 1, & 0\leq t<t_{1},\;\mathrm{takeoff}\;\mathrm{stage}\\ 2, & t_{1}\leq t<t_{2},\;\mathrm{mission}\;\mathrm{execution}\;\mathrm{stage}\\ 3, & t\geq t_{2},\;\mathrm{landing}\;\mathrm{stage} \end{array}\right.$$

To distinguish the physical disturbance torques from their equivalent representations in the angular-velocity dynamics, two notation levels are adopted throughout this section. For each disturbance source i∈{act,res,env,load,ge,dyn}, $\tau_{i}\left(t\right)$ denotes the corresponding disturbance torque at the moment level, whereas $d_{i}\left(t\right)=J^{-1}\tau_{i}\left(t\right)$ denotes the equivalent disturbance entering the angular-velocity dynamic equation. In particular, $\tau_{\mathrm{res}}\left(t\right)$ and $d_{\mathrm{res}}\left(t\right)$ denote the structural-resonance-induced torque and its equivalent contribution to the angular-velocity dynamics, respectively.

According to the mission-stage variable σ(t), the dominant disturbance mechanisms vary among the takeoff, mission execution, and landing stages. The takeoff stage is mainly affected by actuator-output mismatch, payload offset, near-ground aerodynamic effects, local airflow, and structural vibration. During mission execution, actuator faults, payload variation, environmental airflow, unmodeled dynamics, and uncertain resonance modes may act simultaneously. During landing, near-ground aerodynamic effects, recovery-site airflow, actuator faults, residual payload effects, structural vibration, and unmodeled dynamics become dominant. The corresponding stage-dependent lumped disturbances are specified after the physical disturbance models and torque-to-state mapping are established.

The physical sources corresponding to the above equivalent disturbances act on the roll, pitch, and yaw channels through generalized disturbance torques. At the moment level, the total equivalent disturbance torque is defined as

(6) $$\tau_{d}\left(t\right)=\tau_{\mathrm{act}}\left(t\right)+\tau_{\mathrm{res}}\left(t\right)+\tau_{\mathrm{env}}\left(t\right)+\tau_{\mathrm{load}}\left(t\right)+\tau_{\mathrm{ge}}\left(t\right)+\tau_{\mathrm{dyn}}\left(t\right)$$

where

$$\tau_{d}\left(t\right)=\left[\begin{array}{c} \tau_{d\phi }\left(t\right)\\ \tau_{d\theta }\left(t\right)\\ \tau_{d\psi }\left(t\right) \end{array}\right]$$

In (6), $\tau_{\mathrm{act}}\left(t\right)$ denotes the actuator-side mismatch induced by ESC–motor tracking dynamics, control-efficiency loss, actuator bias, and torque saturation; $\tau_{\mathrm{res}}\left(t\right)$ denotes the structural-resonance-induced torque associated with ESC ripple, rotor imbalance, and motor or propeller degradation; $\tau_{\mathrm{env}}\left(t\right)$ denotes the environmental disturbance torque caused by gusts, thermal airflow, building-induced airflow, and dust flow; $\tau_{\mathrm{load}}\left(t\right)$ denotes the torque induced by rescue-payload offset or payload variation; $\tau_{\mathrm{ge}}\left(t\right)$ denotes the near-ground aerodynamic torque during takeoff and landing; and $\tau_{\mathrm{dyn}}\left(t\right)$ denotes the residual torque caused by unmodeled dynamics and parameter uncertainties.

Let $u\left(t\right)\in R^{3}$ denote the commanded attitude-control torque generated online by the closed-loop controller, and let $u_{a}\left(t\right)\in R^{3}$ denote the torque response delivered by the ESC–motor subsystem before the actuator faults are applied. Considering the finite response speed and torque limits of the ESC–motor subsystem, its command-tracking dynamics are represented by

(7) $$T_{\mathrm{esc}}{\dot{u}}_{a}\left(t\right)+u_{a}\left(t\right)={\mathrm{sat}}_{\bar{u}}\left(u(t)\right)$$

where

$$T_{\mathrm{esc}}=\mathrm{diag}\left\{T_{\mathrm{esc},\phi },T_{\mathrm{esc},\theta },T_{\mathrm{esc},\psi }\right\}>0$$

is the equivalent ESC–motor time-constant matrix, and ${\mathrm{sat}}_{\bar{u}}\left(\cdot \right)$ denotes the componentwise actuator-torque saturation with the prescribed torque limit $\bar{u}$. The actuator control-efficiency matrix is defined as

$$E\left(t\right)=\mathrm{diag}\left\{\eta_{\phi }\left(t\right),\eta_{\theta }\left(t\right),\eta_{\psi }\left(t\right)\right\},\;0<\eta_{i}\left(t\right)\leq 1$$

where $\eta_{i}\left(t\right)=1$ represents normal operation and $0<\eta_{i}\left(t\right)<1$ represents a partial loss of control effectiveness in the corresponding attitude channel. Let $b_{u}\left(t\right)\in R^{3}$ denote the additive actuator-bias torque. The torque actually delivered to the rigid-body dynamics is then expressed as

$$\tau_{a}\left(t\right)=E\left(t\right)u_{a}\left(t\right)+b_{u}\left(t\right)$$

Accordingly, the actuator-side fault-disturbance torque relative to the commanded torque u(t) is given by

$$\begin{array}{cc} \tau_{\mathrm{act}}\left(t\right) & =\tau_{a}\left(t\right)-u\left(t\right)\\ & =E\left(t\right)u_{a}\left(t\right)+b_{u}\left(t\right)-u\left(t\right)\\ & =\left(E\left(t\right)-I_{3}\right)u\left(t\right)+E\left(t\right)\left(u_{a}\left(t\right)-u\left(t\right)\right)+b_{u}\left(t\right) \end{array}$$

The first term represents the torque deviation caused by the loss of control effectiveness, the second term describes the ESC–motor command-tracking mismatch, and the third term represents the additive actuator-bias fault. Therefore, the finite ESC response, actuator saturation, efficiency loss, and bias fault are incorporated explicitly into $\tau_{\mathrm{act}}\left(t\right)$ rather than being represented by an additional unspecified ESC disturbance term.

To further characterize the uncertain high-frequency structural resonance induced by ESC ripple, rotor imbalance, and motor or propeller degradation, an equivalent second-order modal model is introduced as

(8) $${\ddot{q}}_{r}\left(t\right)+2Z_{\mathrm{res}}\Omega_{\mathrm{res}}{\dot{q}}_{r}\left(t\right)+\Omega_{\mathrm{res}}^{2}q_{r}\left(t\right)=B_{\mathrm{res}}s_{r}\left(t\right)$$

where $q_{r}\left(t\right)\in R^{3}$ denotes the equivalent structural modal displacement, $s_{r}\left(t\right)\in R^{3}$ denotes the bounded resonance excitation, and

$$\Omega_{\mathrm{res}}=\mathrm{diag}\left\{\omega_{r,\phi },\omega_{r,\theta },\omega_{r,\psi }\right\},$$

$$Z_{\mathrm{res}}=\mathrm{diag}\left\{\zeta_{r,\phi },\zeta_{r,\theta },\zeta_{r,\psi }\right\}$$

contain the modal resonance frequencies and the corresponding damping ratios, respectively. The torque induced by the structural resonance is expressed as

$$\tau_{\mathrm{res}}\left(t\right)=C_{\mathrm{res},q}q_{r}\left(t\right)+C_{\mathrm{res},v}{\dot{q}}_{r}\left(t\right)$$

where $C_{\mathrm{res},q}$ and $C_{\mathrm{res},v}$ map the equivalent modal displacement and velocity into the three-axis attitude-torque channels.

Disturbance-observer-based vibration suppression in rotating electromechanical systems has demonstrated that resonance-induced disturbances and observer bandwidth should be considered jointly in practical implementations [41]. Motivated by this observation, the structural resonance considered in this study is not represented by a single prescribed sinusoidal torque. Instead, its modal frequencies, damping ratios, and excitation amplitudes are allowed to vary within the prescribed ranges $\omega_{r,i}\in \left[{\underline{\omega }}_{r,i},{\bar{\omega }}_{r,i}\right]$, $\zeta_{r,i}\in \left[{\underline{\zeta }}_{r,i},{\bar{\zeta }}_{r,i}\right]$, ${\underline{\zeta }}_{r,i}>0$, and ${∥s_{r}\left(t\right)∥}_{2}\leq {\bar{s}}_{r}$, where i∈{ϕ,θ,ψ}. The resulting resonance-induced contribution to the angular-velocity dynamics is $d_{\mathrm{res}}\left(t\right)=J^{-1}\tau_{\mathrm{res}}\left(t\right)$. The influence of the uncertain resonance frequency, damping ratio, and excitation amplitude on the estimation performance is subsequently evaluated through frequency-sweep and parameter-uncertainty simulations in Section 4.

For the payload-induced torque, the offset between the rescue payload and the UAV center of mass may generate an additional moment. It can be described as

$$\tau_{\mathrm{load}}\left(t\right)=r_{\mathrm{load}}\left(t\right)\times F_{\mathrm{load}}\left(t\right)$$

where $r_{\mathrm{load}}\left(t\right)$ denotes the position vector from the UAV center of mass to the payload center, and $F_{\mathrm{load}}\left(t\right)$ denotes the equivalent force caused by payload weight, payload variation, or payload release.

For the near-ground aerodynamic effect during takeoff and landing, a height-dependent activation function is introduced as

(9) $$\alpha_{g}\left(h\right)=\frac{1}{2}\left[1-\tanh\left(\frac{h\left(t\right)-h_{g}}{T_{h}}\right)\right]$$

where h(t) denotes the flight height, $h_{g}$ denotes the height threshold at which the near-ground effect becomes significant, and $T_{h}>0$ is a smoothing parameter. Then, the near-ground aerodynamic torque can be expressed as

(10) $$\tau_{\mathrm{ge}}\left(t\right)=\alpha_{g}\left(h\right){\bar{\tau }}_{\mathrm{ge}}\left(t\right)$$

where ${\bar{\tau }}_{\mathrm{ge}}\left(t\right)$ denotes a bounded and possibly time-varying nominal near-ground aerodynamic torque rather than a static aerodynamic coefficient.

The height-dependent function $\alpha_{g}\left(h\right)$ is introduced only as a smooth activation envelope describing the increasing relevance of near-ground aerodynamic effects as the UAV approaches the ground; it is not intended to constitute a high-fidelity aerodynamic model of rotor wakes or pressure-cushion dynamics. In a practical takeoff or landing process, the actual near-ground aerodynamic torque may additionally depend on the vertical velocity, rotor rotational speeds, instantaneous attitude, ground-surface characteristics, and unsteady wake interactions. It may therefore be conceptually decomposed as

$$\tau_{\mathrm{ge}}^{\mathrm{real}}\left(t\right)=\alpha_{g}\left(h\right){\bar{\tau }}_{\mathrm{ge}}\left(t\right)+\tau_{\mathrm{ge},\mathrm{res}}\left(h,\dot{h},\Omega_{\mathrm{rot}},x_{1},t\right)$$

where $\Omega_{\mathrm{rot}}$ denotes the rotor-speed vector, and $\tau_{\mathrm{ge},\mathrm{res}}$ collects the higher-order and unsteady aerodynamic effects that are not explicitly described by the smooth height-dependent envelope. When this residual torque is bounded and its principal frequency components lie within the considered modeling and measurement bandwidths, it can be incorporated into the unmodeled-dynamics torque $\tau_{\mathrm{dyn}}\left(t\right)$. After the inertia mapping, its equivalent influence is consequently included in $d_{\mathrm{dyn}}\left(t\right)$ and the lumped fault disturbance Δ(t). Related studies of hybrid VTOL UAVs under transition flight have also emphasized the importance of accounting for pronounced aerodynamic variations, model uncertainties, and multiple fault effects during flight-mode transitions [42]. When the residual near-ground torque is bounded, its equivalent influence can be incorporated into $\tau_{\mathrm{dyn}}\left(t\right)$. The present low-order near-ground model does not explicitly resolve the complete broadband wake-vortex and pressure-cushion dynamics; however, their bounded residual effects are retained in the lumped disturbance. This aerodynamic residual is distinguished from the actuator- and structure-induced resonance modes explicitly represented by $\tau_{\mathrm{res}}\left(t\right)$.

The environmental disturbance torque and the residual unmodeled dynamic torque are generally difficult to model accurately under emergency rescue mission conditions. Therefore, $\tau_{\mathrm{env}}\left(t\right)$ and $\tau_{\mathrm{dyn}}\left(t\right)$ are treated as bounded time-varying equivalent torques. Together with the actuator-side fault-disturbance torque, uncertain structural-resonance-induced torque, payload-induced torque, and near-ground aerodynamic torque, these terms constitute the total disturbance torque $\tau_{d}\left(t\right)$ defined in (6), thereby providing a unified representation of the multi-source fault and disturbance effects encountered during the takeoff, mission execution, and landing stages.

Accordingly, the rotational dynamics in the angular-velocity channel can be written as

(11) $$J{\dot{x}}_{2}\left(t\right)=-x_{2}\left(t\right)\times Jx_{2}\left(t\right)+u\left(t\right)+\tau_{d}\left(t\right)$$

where $u\left(t\right)={\left[\tau_{\phi }\left(t\right),\tau_{\theta }\left(t\right),\tau_{\psi }\left(t\right)\right]}^{T}$ is the commanded attitude-control torque supplied to the ESC–actuator subsystem rather than the torque actually delivered by the actuators. The total disturbance torque $\tau_{d}\left(t\right)$ represents the difference between the nominal commanded torque and the actual torque acting on the rigid-body dynamics, together with the external and unmodeled disturbance torques. Since $\tau_{\mathrm{act}}\left(t\right)=\tau_{a}\left(t\right)-u\left(t\right)$, the combination $u\left(t\right)+\tau_{\mathrm{act}}\left(t\right)$ is equal to the actual actuator torque $\tau_{a}\left(t\right)$; therefore, the actuator output is not counted twice in the above dynamics.

The equivalent lumped fault disturbance entering the angular-velocity state equation is then defined as

(12) $$\begin{array}{cc} \Delta (t) & =J^{-1}\tau_{d}\left(t\right)\\ \; & =d_{\mathrm{act}}\left(t\right)+d_{\mathrm{res}}\left(t\right)+d_{\mathrm{env}}\left(t\right)+d_{\mathrm{load}}\left(t\right)+d_{\mathrm{ge}}\left(t\right)+d_{\mathrm{dyn}}\left(t\right) \end{array}$$

where $d_{i}\left(t\right)=J^{-1}\tau_{i}\left(t\right)$, i∈{act,res,env,load,ge,dyn}. In particular, $d_{\mathrm{res}}\left(t\right)=J^{-1}\tau_{\mathrm{res}}\left(t\right)$ denotes the equivalent contribution of the uncertain structural resonance to the angular-velocity dynamics.

The disturbance components that are inactive in a particular mission stage are taken as zero. Accordingly, the angular-velocity dynamics with multi-source fault disturbances can be expressed in the following affine form:

$${\dot{x}}_{2}=f\left(x\right)+gu+\Delta \left(t\right)$$

where

$$f\left(x\right)=J^{-1}\left(-x_{2}\times Jx_{2}\right)\;g=J^{-1}$$

If the rotational inertia matrix is given by

$$J=\mathrm{diag}\{J_{x},J_{y},J_{z}\}$$

and the total equivalent disturbance torque is denoted as

$$\tau_{d}\left(t\right)=\left[\begin{array}{c} \tau_{d\phi }\left(t\right)\\ \tau_{d\theta }\left(t\right)\\ \tau_{d\psi }\left(t\right) \end{array}\right]$$

then the lumped fault disturbance in the angular-velocity state channel can be further written as

(13) $$\Delta \left(t\right)=\left[\begin{array}{c} \Delta_{\phi }\left(t\right)\\ \Delta_{\theta }\left(t\right)\\ \Delta_{\psi }\left(t\right) \end{array}\right]=\left[\begin{array}{c} \frac{\tau_{d\phi }\left(t\right)}{J_{x}}\\ \frac{\tau_{d\theta }\left(t\right)}{J_{y}}\\ \frac{\tau_{d\psi }\left(t\right)}{J_{z}} \end{array}\right]$$

In this way, the actuator-side mismatch, uncertain structural resonance, environmental disturbances, payload effects, near-ground aerodynamic effects, and unmodeled dynamics generated during the three mission stages are uniformly mapped into the angular-velocity state channel as the lumped fault disturbance Δ(t) defined in Equation (12).

According to the mission-stage variable σ(t), the lumped fault disturbance entering the angular-velocity state channel can be written in the following stage-dependent form:

$$\Delta \left(t\right)=\left\{\begin{array}{cc} \Delta_{\mathrm{to}}\left(t\right), & \sigma (t)=1\\ \Delta_{\mathrm{mis}}\left(t\right), & \sigma (t)=2\\ \Delta_{\mathrm{land}}\left(t\right), & \sigma (t)=3 \end{array}\right.$$

Specifically, in the takeoff stage, the lumped fault disturbance is given by

$$\Delta_{\mathrm{to}}\left(t\right)=d_{\mathrm{act}}\left(t\right)+d_{\mathrm{res}}\left(t\right)+d_{\mathrm{load}}\left(t\right)+d_{\mathrm{ge},\mathrm{to}}\left(t\right)+d_{\mathrm{env},\mathrm{to}}\left(t\right)$$

where $d_{\mathrm{act}}\left(t\right)$ denotes the equivalent disturbance induced by actuator faults, $d_{\mathrm{load}}\left(t\right)$ denotes the equivalent disturbance induced by rescue-payload variations, $d_{\mathrm{res}}\left(t\right)$ denotes the equivalent disturbance induced by the uncertain structural resonance associated with ESC ripple, rotor imbalance, or actuator degradation, $d_{\mathrm{ge},\mathrm{to}}\left(t\right)$ denotes the equivalent disturbance induced by the near-ground aerodynamic effect during takeoff, and $d_{\mathrm{env},\mathrm{to}}\left(t\right)$ denotes the equivalent disturbance induced by the local environmental airflow around the temporary takeoff site.

In the mission execution stage, the lumped fault disturbance is described as

$$\Delta_{\mathrm{mis}}\left(t\right)=d_{\mathrm{act}}\left(t\right)+d_{\mathrm{res}}\left(t\right)+d_{\mathrm{load}}\left(t\right)+d_{\mathrm{env},\mathrm{mis}}\left(t\right)+d_{\mathrm{dyn}}\left(t\right)$$

where $d_{\mathrm{env},\mathrm{mis}}\left(t\right)$ represents the equivalent disturbance induced by gusts, thermal airflow, building-induced airflow, and dust flow in the disaster environment, and $d_{\mathrm{dyn}}\left(t\right)$ represents the equivalent disturbance induced by unmodeled dynamics and parameter uncertainties.

In the landing stage, the lumped fault disturbance is expressed as

$$\Delta_{\mathrm{land}}\left(t\right)=d_{\mathrm{act}}\left(t\right)+d_{\mathrm{res}}\left(t\right)+d_{\mathrm{load}}\left(t\right)+d_{\mathrm{ge},\mathrm{land}}\left(t\right)+d_{\mathrm{env},\mathrm{land}}\left(t\right)+d_{\mathrm{dyn}}\left(t\right)$$

where $d_{\mathrm{ge},\mathrm{land}}\left(t\right)$ denotes the equivalent disturbance induced by the near-ground aerodynamic effect during landing, and $d_{\mathrm{env},\mathrm{land}}\left(t\right)$ denotes the equivalent disturbance induced by the environmental airflow around the temporary recovery area.

### 2.3. Formulation of the Multi-Stage Fault-Disturbance Estimation and Closed-Loop Compensation Problem

Based on the nominal quadrotor attitude dynamics established in Section 2.1 and the multi-stage lumped fault-disturbance model presented in Section 2.2, this subsection formulates the fixed-time estimation problem and its closed-loop compensation framework. The proposed estimator remains the main methodological contribution, while a baseline attitude controller and a bandwidth-limited disturbance-compensation channel are introduced to evaluate the influence of the estimation error and estimation lag on the augmented closed-loop system.

By incorporating the lumped fault disturbance Δ(t) into the angular-velocity dynamic channel while retaining the complete attitude kinematic relationship, the attitude system of the emergency rescue quadrotor UAV can be expressed as

(14) $$\left\{\begin{array}{cc} {\dot{x}}_{1} & =W\left(x_{1}\right)x_{2},\\ {\dot{x}}_{2} & =f(x)+gu+\Delta (t) \end{array}\right.$$

For the prescribed structural-resonance frequency range, the modal frequencies and excitation amplitudes are bounded, while the damping ratios are bounded away from zero, namely

$$\begin{array}{cc} 0 & <{\underline{\omega }}_{r,i}\leq \omega_{r,i}\leq {\bar{\omega }}_{r,i}<\infty \\ 0 & <{\underline{\zeta }}_{r,i}\leq \zeta_{r,i}\leq {\bar{\zeta }}_{r,i},\\ ∥s_{r}\left(t\right)∥ & \leq {\bar{s}}_{r}\;i\in \left\{\phi ,\theta ,\psi \right\} \end{array}$$

Therefore, the resonance-induced torque and its variation rate remain bounded within each fixed mission stage.

**Assumption** **1.** *For each fixed mission stage k∈{1,2,3}, where σ(t)=k, the lumped fault disturbance Δ(t) is continuous and differentiable, and there exists a positive constant ${\bar{\rho }}_{k}>0$ such that*

$${∥\dot{\Delta }\left(t\right)∥}_{2}\leq {\bar{\rho }}_{k},\;\sigma \left(t\right)=k$$

*At the stage transition instants $t_{1}$ and $t_{2}$, the lumped fault disturbance is allowed to have bounded jumps. That is, there exist positive constants ${\bar{J}}_{1}>0$ and ${\bar{J}}_{2}>0$ such that*

$$∥\Delta \left(t_{i}^{+}\right)-\Delta \left(t_{i}^{-}\right)∥\leq {\bar{J}}_{i},\;i=1,2$$

*Furthermore, the lumped fault disturbance itself is assumed to be bounded during the whole takeoff–mission–landing process. Namely, there exists a positive constant $\bar{\Delta }>0$ such that*

$$∥\Delta (t)∥\leq \bar{\Delta },\;t\geq 0$$

*The bounds ${\bar{\rho }}_{k}$ and $\bar{\Delta }$ include the effects of the ESC–motor tracking mismatch and the uncertain structural resonance within the prescribed frequency range.*

The above assumptions indicate that the disturbance is stage-dependent and time-varying, but its variation rate and possible jumps at the stage transition instants are bounded. These properties provide the basis for the fixed-time estimation of Δ(t) in the following section.

**Figure 1:**
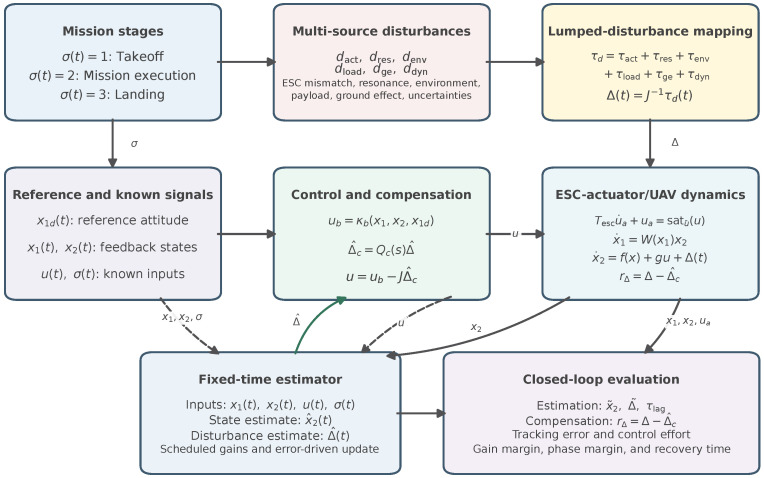
Closed-loop framework for multi-stage fault-disturbance estimation and bandwidth-limited compensation in emergency rescue UAV missions.

As shown in Figure 1, the revised framework consists of multi-stage disturbance modeling, a baseline closed-loop attitude controller, ESC–actuator dynamics, fixed-time fault-disturbance estimation, and a bandwidth-limited disturbance-compensation channel. The actuator-side mismatch, structural resonance, environmental airflow, payload effects, near-ground aerodynamic effects, and unmodeled dynamics are uniformly mapped into the angular-velocity dynamic channel as the lumped disturbance $\Delta \left(t\right)=J^{-1}\tau_{d}\left(t\right)$. Based on the available signals $x_{1}\left(t\right)$, $x_{2}\left(t\right)$, u(t), and σ(t), the estimator generates ${\hat{x}}_{2}\left(t\right)$ and $\hat{\Delta }\left(t\right)$. The processed disturbance estimate is then incorporated into the commanded torque to evaluate the closed-loop influence of the estimation error and estimation lag.

In system (14), $x_{1}\left(t\right)={\left[\phi \left(t\right),\theta \left(t\right),\psi \left(t\right)\right]}^{T}$ denotes the attitude-angle vector, and $x_{2}\left(t\right)={\left[p\left(t\right),q\left(t\right),r\left(t\right)\right]}^{T}$ denotes the body angular-velocity vector. The nominal torque generated online by the baseline attitude controller is expressed as

$$u_{b}\left(t\right)=\kappa_{b}\left(x_{1}\left(t\right),x_{2}\left(t\right),x_{1d}\left(t\right)\right)$$

where $x_{1d}\left(t\right)$ is the reference attitude and $\kappa_{b}\left(\cdot \right)$ represents the baseline control law. To evaluate the closed-loop applicability of the proposed estimator, an estimator-based disturbance-compensation torque $u_{\mathrm{comp}}\left(t\right)$ is added to the nominal control torque. Therefore, the total commanded torque supplied to the ESC–actuator subsystem is defined as

$$u\left(t\right)=u_{b}\left(t\right)+u_{\mathrm{comp}}\left(t\right)$$

The commanded torque u(t) is distinguished from the actual actuator torque $u_{a}\left(t\right)$ generated by the ESC–motor subsystem. Owing to the finite ESC bandwidth, motor–propeller dynamics, and actuator saturation, $u_{a}\left(t\right)$ may not instantaneously coincide with u(t). The equivalent effect of this tracking mismatch, $g\left(x\right)\left[u_{a}\left(t\right)-u\left(t\right)\right]$, is incorporated into the lumped fault disturbance Δ(t).

The term Δ(t) represents the lumped fault disturbance entering the angular-velocity dynamic channel. The control-dependent and additive fault terms introduced in system (4) are incorporated through $g\left(x\right)u_{\mathrm{fault}}\left(t\right)+d_{\mathrm{fault}}\left(t\right)$. Together with these fault effects, Δ(t) includes the equivalent effects of the ESC–motor tracking mismatch, actuator efficiency loss, actuator bias, uncertain structural resonance within the prescribed frequency range, environmental airflow, rescue-payload variation, near-ground aerodynamic effects, and residual unmodeled dynamics during the takeoff, mission execution, and landing stages.

Although the proposed fixed-time estimator remains the main methodological contribution of this study, its applicability is evaluated within a closed-loop attitude-control environment. Directly feeding the raw disturbance estimate $\hat{\Delta }\left(t\right)$ into the actuator system may amplify measurement noise and excite the uncertain high-frequency structural modes. Therefore, the raw disturbance estimate is processed by a stable bandwidth-limited compensation filter $Q_{c}\left(s\right)$, yielding

$${\hat{\Delta }}_{c}\left(s\right)=Q_{c}\left(s\right)\hat{\Delta }\left(s\right)$$

The filter $Q_{c}\left(s\right)$ is selected as a stable and strictly proper transfer matrix with approximately unit gain in the low-frequency disturbance-compensation range and sufficient attenuation in the prescribed high-frequency resonance range. Its bandwidth is chosen to retain the dominant low- and medium-frequency fault-disturbance components while suppressing structural-resonance components and measurement noise that should not be directly fed back to the actuator. The filter bandwidth and the required resonance-attenuation parameters are specified in Section 4, and the additional phase lag introduced by $Q_{c}\left(s\right)$ is included in the subsequent finite-band closed-loop sensitivity analysis.

Since $g\left(x\right)=J^{-1}$, the estimator-based compensation torque is defined as

$$u_{\mathrm{comp}}\left(t\right)=-g^{-1}\left(x\right){\hat{\Delta }}_{c}\left(t\right)=-J{\hat{\Delta }}_{c}\left(t\right)$$

Accordingly, the total commanded torque supplied to the ESC–actuator subsystem becomes

$$u\left(t\right)=u_{b}\left(t\right)+u_{\mathrm{comp}}\left(t\right)=u_{b}\left(t\right)-J{\hat{\Delta }}_{c}\left(t\right)$$

Substituting the total commanded torque into system (14) gives

$$\begin{array}{cc} {\dot{x}}_{2}\left(t\right) & =f\left(x\right)+g\left(x\right)\left[u_{b}\left(t\right)-J{\hat{\Delta }}_{c}\left(t\right)\right]+\Delta \left(t\right)\\ & =f\left(x\right)+g\left(x\right)u_{b}\left(t\right)+\Delta \left(t\right)-{\hat{\Delta }}_{c}\left(t\right) \end{array}$$

The residual disturbance actually acting on the compensated closed-loop angular-velocity channel is therefore defined as

(15) $$r_{\Delta }\left(t\right)=\Delta \left(t\right)-{\hat{\Delta }}_{c}\left(t\right)$$

and the compensated angular-velocity dynamics can be written as

(16) $${\dot{x}}_{2}\left(t\right)=f\left(x\right)+g\left(x\right)u_{b}\left(t\right)+r_{\Delta }\left(t\right)$$

Therefore, after the estimator output is incorporated into the compensation channel, the augmented closed-loop system in (16) is driven by the residual disturbance $r_{\Delta }\left(t\right)$ defined in (15) rather than by the complete lumped fault disturbance Δ(t). The residual disturbance contains both the raw estimation error and the additional amplitude and phase mismatch introduced by the bandwidth-limited compensation filter.

To formulate the closed-loop stability condition associated with the residual disturbance, define the attitude-tracking-error vector as

$$e_{c}\left(t\right)={\left[\begin{array}{cc} e_{1}^{T}\left(t\right) & e_{2}^{T}\left(t\right) \end{array}\right]}^{T}$$

where

$$e_{1}\left(t\right)=x_{1}\left(t\right)-x_{1d}\left(t\right),\;e_{2}\left(t\right)=x_{2}\left(t\right)-x_{2d}\left(t\right)$$

Here, $x_{1d}\left(t\right)$ denotes the reference attitude, and $x_{2d}\left(t\right)$ denotes the corresponding desired body angular velocity generated by the baseline attitude-control system.

**Assumption** **2.** *The nominal closed-loop attitude system under the baseline controller $u_{b}\left(t\right)$ is locally asymptotically stable when $r_{\Delta }\left(t\right)=0$. Moreover, there exist a neighborhood $D_{c}$ of the equilibrium $e_{c}=0$, a continuously differentiable positive-definite Lyapunov function $V_{c}\left(e_{c}\right)$, and positive constants ${\underline{c}}_{c}$, ${\bar{c}}_{c}$, $\alpha_{c}$, and $\beta_{c}$ such that, for all $e_{c}\in D_{c}$,*

$${\underline{c}}_{c}{∥e_{c}∥}^{2}\leq V_{c}\left(e_{c}\right)\leq {\bar{c}}_{c}{∥e_{c}∥}^{2}$$

*and*

$${\dot{V}}_{c}\left(e_{c}\right)\leq -\alpha_{c}{∥e_{c}∥}^{2}+\beta_{c}∥e_{c}∥∥r_{\Delta }∥$$

Assumption 2 provides an input-to-state-stability-type condition for the baseline closed-loop attitude system with respect to the residual disturbance $r_{\Delta }\left(t\right)$. By applying Young’s inequality, the derivative of $V_{c}$ satisfies

$${\dot{V}}_{c}\left(e_{c}\right)\leq -\frac{\alpha_{c}}{2}{∥e_{c}∥}^{2}+\frac{\beta_{c}^{2}}{2\alpha_{c}}{∥r_{\Delta }∥}^{2}$$

Therefore, a bounded residual disturbance does not destroy the local boundedness of the augmented closed-loop attitude system. The corresponding ultimate bound of the tracking error, together with its dependence on the fault-disturbance estimation and compensation errors, is established after the fixed-time convergence result of the proposed estimator.

To facilitate the subsequent estimator design, the estimate of the state is denoted by ${\hat{x}}_{2}\left(t\right)$, and the estimate of the lumped fault disturbance is denoted by $\hat{\Delta }\left(t\right)$. Then, the state estimation error and the lumped fault-disturbance estimation error are defined as follows:

$${\tilde{x}}_{2}\left(t\right)=x_{2}\left(t\right)-{\hat{x}}_{2}\left(t\right)$$

$$\tilde{\Delta }\left(t\right)=\Delta \left(t\right)-\hat{\Delta }\left(t\right)$$

where ${\tilde{x}}_{2}\left(t\right)$ represents the state estimation error, and $\tilde{\Delta }\left(t\right)$ represents the estimation error of the lumped fault disturbance.

It should be noted that $\tilde{\Delta }\left(t\right)$ denotes the raw fault-disturbance estimation error used in the estimator convergence analysis, whereas $r_{\Delta }\left(t\right)=\Delta \left(t\right)-{\hat{\Delta }}_{c}\left(t\right)$, as defined in (15), denotes the residual disturbance acting on the compensated closed-loop system. These two quantities are generally different because of the bandwidth-limited compensation filter $Q_{c}\left(s\right)$.

The objective of the proposed estimator is to reconstruct the lumped fault disturbance Δ(t) defined in Equation (12) in each fixed mission stage. For every k∈{1,2,3}, the state estimation error ${\tilde{x}}_{2}\left(t\right)$ and the lumped fault-disturbance estimation error $\tilde{\Delta }\left(t\right)$ should enter and remain in the corresponding bounded neighborhoods $\Omega_{x,k}$ and $\Omega_{\Delta ,k}$ within a fixed time independent of the initial estimation errors, namely

$$\begin{array}{cc} {\tilde{x}}_{2}\left(t\right) & \in \Omega_{x,k},\;\tilde{\Delta }\left(t\right)\in \Omega_{\Delta ,k}\\ t & \in \left[t_{k-1}+T_{z,k},t_{k}\right),\;\sigma \left(t\right)=k \end{array}$$

At a stage-transition instant, the bounded jump of Δ(t) may temporarily drive the estimation errors outside the preceding-stage neighborhoods. Nevertheless, the errors should remain bounded and re-enter the new-stage neighborhoods within a uniformly bounded recovery time.

Throughout the takeoff–mission execution–landing process, the estimator is required to reconstruct the stage-dependent lumped disturbance containing ESC–actuator mismatch, actuator faults, uncertain structural resonance, rescue-payload variation, environmental airflow, near-ground aerodynamic effects, and residual unmodeled dynamics. Within each fixed mission stage, the estimator should track both the slowly varying disturbance components and the prescribed-band resonance-induced components while maintaining bounded estimation errors. Following a bounded disturbance jump at a stage transition, the estimator should recover within a uniformly bounded time. Since the disturbance estimate is subsequently incorporated into the compensation channel, the estimation lag and the additional phase introduced by $Q_{c}\left(s\right)$ should also be quantified and verified so as not to destroy the stability margins of the augmented closed-loop system.

Therefore, under the conditions that $x_{1}\left(t\right)$, $x_{2}\left(t\right)$, u(t), and σ(t) are available, the problem addressed in this study is to design a stage-dependent fixed-time estimator such that the estimation errors ${\tilde{x}}_{2}\left(t\right)$ and $\tilde{\Delta }\left(t\right)$ achieve stage-wise practical fixed-time convergence, the residual disturbance $r_{\Delta }\left(t\right)$ defined in (15) remains bounded after estimator-based compensation, and the augmented closed-loop attitude system maintains stable tracking behavior under the considered ESC dynamics, structural resonance, estimation lag, measurement noise, and stage-transition conditions.

The present work does not propose a new fault-tolerant controller. Instead, a conventional baseline attitude controller and an estimator-based compensation channel are employed to evaluate the closed-loop applicability of the proposed estimator. Theoretical boundedness with respect to the residual disturbance under Assumption 2 is considered in the present study, whereas systematic observer–controller co-design and experimental flight validation remain subjects of future work.

## 3. Main Results

In this section, a stage-dependent fixed-time fault-disturbance estimator is developed for the problem formulated in Section 2.3. The lumped disturbance Δ(t) includes the equivalent effects of ESC–motor tracking mismatch, actuator efficiency loss, actuator bias, uncertain structural resonance within the prescribed frequency range, environmental airflow, rescue-payload variation, near-ground aerodynamic effects, and residual unmodeled dynamics. Although these components originate from different physical mechanisms, they are uniformly represented in the angular-velocity dynamic channel.

The estimator is designed to achieve practical fixed-time convergence within each fixed mission stage and to recover after bounded disturbance jumps at the stage-transition instants. The resulting disturbance estimate is subsequently processed by the bandwidth-limited compensation channel introduced in Section 2.3. Therefore, this section first establishes the stage-wise fixed-time estimation result and then derives the boundedness implication for the augmented closed-loop system under the residual disturbance $r_{\Delta }\left(t\right)$ defined in Equation (15). The overall closed-loop estimation and compensation framework is shown in Figure 1.

The state estimation error is defined as ${\tilde{x}}_{2}=x_{2}-{\hat{x}}_{2}$, and the lumped fault-disturbance estimation error is defined as $\tilde{\Delta }=\Delta -\hat{\Delta }$. Then, the fault estimator is designed as follows:

(17) $$\left\{\begin{array}{cc} {\dot{\hat{x}}}_{2} & =f\left(x\right)+gu+\hat{\Delta }+\Lambda_{1}^{\left(\sigma \right)}\Phi_{m_{1}}\;\left({\tilde{x}}_{2}\right)+M_{1}^{\left(\sigma \right)}\Phi_{n_{1}}\;\left({\tilde{x}}_{2}\right)\\ \dot{\hat{\Delta }} & =\Lambda_{2}^{\left(\sigma \right)}\Phi_{m_{2}}\;\left({\tilde{x}}_{2}\right)+M_{2}^{\left(\sigma \right)}\Phi_{n_{2}}\;\left({\tilde{x}}_{2}\right)+\Gamma^{\left(\sigma \right)}\tanh\;\left(\frac{{\tilde{x}}_{2}}{\varepsilon_{e}}\right) \end{array}\right.$$

where the nonlinear signed-power mapping is defined componentwise as $\Phi_{\alpha }\left(z\right)={\left|z\right|}^{\alpha }⊙\mathrm{sgn}\left(z\right)$, with $\Phi_{\alpha }\left(0\right)=0$, and ⊙ denotes the Hadamard product. Since all the power exponents used in the estimator are positive, the signed-power mappings are continuous at the origin. The low-power correction terms $\Phi_{m_{1}}\left({\tilde{x}}_{2}\right)$ and $\Phi_{m_{2}}\left({\tilde{x}}_{2}\right)$, with $0<m_{2}<m_{1}<1$, provide the negative-degree homogeneous behavior required for rapid convergence near the origin. The high-power correction terms $\Phi_{n_{1}}\left({\tilde{x}}_{2}\right)$ and $\Phi_{n_{2}}\left({\tilde{x}}_{2}\right)$, with $n_{2}>n_{1}>1$, provide the positive-degree homogeneous behavior required to obtain a convergence-time bound independent of large initial errors. In addition, $\varepsilon_{e}>0$ is a smoothing parameter, and the hyperbolic tangent function is evaluated componentwise. The bounded smooth injection term $\Gamma^{\left(\sigma \right)}\tanh\;\left({\tilde{x}}_{2}/\varepsilon_{e}\right)$ is introduced to improve the estimation of the time-varying lumped fault disturbance while avoiding a discontinuous switching term in the disturbance-estimation channel.

It should be emphasized that the estimator employs the same total commanded torque $u\left(t\right)=u_{b}\left(t\right)-J{\hat{\Delta }}_{c}\left(t\right)$ as that appearing in the plant model. Therefore, the known term f(x)+gu cancels when the estimation-error dynamics are derived. The ESC–motor tracking mismatch, actuator efficiency loss, actuator bias, and structural-resonance effects remain included in Δ(t). Consequently, the estimator-based closed-loop compensation does not alter the structural form of the estimation-error system provided that the bounds specified in Assumption 1 hold along the closed-loop trajectories.

In practical implementation, the mission-stage variable σ(t) is not estimated by the proposed fault-disturbance estimator. Instead, it is provided as an available scheduling signal by the high-level mission-management module or the flight-control state machine. Recent studies on emergency scheduling have also highlighted the uncertainty of high-level scheduling decisions under unpredictable maneuvering conditions [43]. In a digital implementation, the mission-stage logic is evaluated at discrete sampling instants, and the active gain matrices are selected from a precomputed stage-dependent gain table according to the current value of σ(t). Hysteresis, a confirmation time window, or a prescribed minimum dwell time can be incorporated into the mission-management logic to prevent repeated switching caused by measurement noise or temporary ambiguity near a stage boundary.

When a mission-stage transition occurs, the estimator states are not reset; only the active gain set is updated. Therefore,

$${\hat{x}}_{2}\left(t_{i}^{+}\right)={\hat{x}}_{2}\left(t_{i}^{-}\right),\;\hat{\Delta }\left(t_{i}^{+}\right)=\hat{\Delta }\left(t_{i}^{-}\right)$$

Hence, the gain update does not introduce an impulsive jump into the estimator states themselves, although it may cause a finite change in the estimator right-hand side and state derivatives. Since the scheduled gain matrices, estimation errors, and smooth compensation terms are bounded, this derivative variation remains finite. The ideal hard switching adopted in the analysis should therefore be interpreted as piecewise-constant software gain scheduling implemented at discrete sampling instants rather than as an infinite-bandwidth physical actuation process. In an embedded implementation, hysteresis, minimum-dwell-time logic, numerical saturation, and, if separately verified, a rate-limited or bumpless gain update can be employed to reduce repeated switching and derivative transients. The latter option requires additional stability verification for the intermediate gains and is not covered by the present hard-switching stability result.

In the revised closed-loop framework, the continuous estimate $\hat{\Delta }\left(t\right)$ is processed by the stable compensation filter $Q_{c}\left(s\right)$, whose coefficients and internal states are not switched or reset at mission-stage transitions. Therefore,

$$\begin{array}{cc} {\hat{\Delta }}_{c}\;\left(t_{i}^{+}\right) & ={\hat{\Delta }}_{c}\;\left(t_{i}^{-}\right),\\ u_{\mathrm{comp}}\;\left(t_{i}^{+}\right) & =u_{\mathrm{comp}}\;\left(t_{i}^{-}\right) \end{array}$$

Hence, the discrete update of the estimator gain set does not directly generate an impulsive jump in the compensation torque. It may nevertheless cause a finite change in the derivatives of $\hat{\Delta }\left(t\right)$, ${\hat{\Delta }}_{c}\left(t\right)$, and $u_{\mathrm{comp}}\left(t\right)$. These derivative transients are attenuated by the bandwidth-limited compensation filter and the ESC–motor dynamics.

$$\begin{array}{cc} \Lambda_{1}^{\left(\sigma \right)} & =\mathrm{diag}\left\{\lambda_{11}^{\left(\sigma \right)},\lambda_{12}^{\left(\sigma \right)},\lambda_{13}^{\left(\sigma \right)}\right\}\\ M_{1}^{\left(\sigma \right)} & =\mathrm{diag}\left\{\mu_{11}^{\left(\sigma \right)},\mu_{12}^{\left(\sigma \right)},\mu_{13}^{\left(\sigma \right)}\right\}\\ \Lambda_{2}^{\left(\sigma \right)} & =\mathrm{diag}\left\{\lambda_{21}^{\left(\sigma \right)},\lambda_{22}^{\left(\sigma \right)},\lambda_{23}^{\left(\sigma \right)}\right\}\\ M_{2}^{\left(\sigma \right)} & =\mathrm{diag}\left\{\mu_{21}^{\left(\sigma \right)},\mu_{22}^{\left(\sigma \right)},\mu_{23}^{\left(\sigma \right)}\right\}\\ \Gamma^{\left(\sigma \right)} & =\mathrm{diag}\left\{\gamma_{1}^{\left(\sigma \right)},\gamma_{2}^{\left(\sigma \right)},\gamma_{3}^{\left(\sigma \right)}\right\} \end{array}$$

They satisfy the following parameter conditions:

$$\begin{array}{cc} & \frac{1}{2}<m_{1}<1,\;n_{1}>1,\\ & m_{2}=2m_{1}-1,\;n_{2}=2n_{1}-1,\;\gamma_{i}\left(\sigma \right)>0,\;i=1,2,3,\;\sigma \in \left\{1,2,3\right\} \end{array}$$

Because the plant and the estimator use the same commanded torque u(t), the known control-dependent terms cancel. Therefore, the estimation-error system retains the following form after the estimator output is incorporated into the closed-loop compensation channel. According to (17) and the definitions of the estimation errors, the error system can be expressed as

(18) $$\left\{\begin{array}{cc} {\dot{\tilde{x}}}_{2} & =\tilde{\Delta }-\Lambda_{1}^{\left(\sigma \right)}\Phi_{m_{1}}\;\left({\tilde{x}}_{2}\right)-M_{1}^{\left(\sigma \right)}\Phi_{n_{1}}\;\left({\tilde{x}}_{2}\right)\\ \dot{\tilde{\Delta }} & =\dot{\Delta }-\Lambda_{2}^{\left(\sigma \right)}\Phi_{m_{2}}\;\left({\tilde{x}}_{2}\right)-M_{2}^{\left(\sigma \right)}\Phi_{n_{2}}\;\left({\tilde{x}}_{2}\right)-\Gamma^{\left(\sigma \right)}\tanh\;\left(\frac{{\tilde{x}}_{2}}{\varepsilon_{e}}\right) \end{array}\right.$$

For each fixed mission stage k∈{1,2,3}, define the low-power and high-power gain-induced matrices as

$$R_{1}^{\left(k\right)}=\left[\begin{array}{cc} -\Lambda_{1}^{\left(k\right)} & I_{3}\\ -\Lambda_{2}^{\left(k\right)} & 0 \end{array}\right],\;R_{2}^{\left(k\right)}=\left[\begin{array}{cc} -M_{1}^{\left(k\right)} & I_{3}\\ -M_{2}^{\left(k\right)} & 0 \end{array}\right]$$

For each fixed mission stage k∈{1,2,3}, the stage-dependent estimator gains are selected such that $R_{1}^{\left(k\right)}$ and $R_{2}^{\left(k\right)}$ are Hurwitz and admit a common quadratic Lyapunov matrix. More precisely, there exist symmetric positive-definite matrices $P_{k}$, $Q_{1k}$, and $Q_{2k}$ satisfying

(19) $$\left\{\begin{array}{cc} {\left(R_{1}^{\left(k\right)}\right)}^{T}P_{k}+P_{k}R_{1}^{\left(k\right)} & ⪯-Q_{1k}\\ {\left(R_{2}^{\left(k\right)}\right)}^{T}P_{k}+P_{k}R_{2}^{\left(k\right)} & ⪯-Q_{2k} \end{array}\right.$$

These inequalities provide a stage-wise common quadratic stability condition for the low-power and high-power limiting error systems. They can be verified directly after the stage-dependent estimator gains have been specified.

**Lemma** **1**  ([44])**.** *Let V(x) be a positive definite and differentiable function. For positive constants $a_{1}$, $a_{2}$, c and exponents $0<\rho_{1}<1$, $\rho_{2}>1$, assume that, along the system trajectory, the following inequality holds:*

$$\dot{V}\left(x\right)\leq -a_{1}V^{\rho_{1}}\left(x\right)-a_{2}V^{\rho_{2}}\left(x\right)+c$$

*and then, for any selected ϑ∈(0,1), define $\bar{V}>0$ satisfying*

$$a_{1}{\bar{V}}^{\rho_{1}}+a_{2}{\bar{V}}^{\rho_{2}}=\frac{c}{1-\vartheta }$$

*Then the set $\Omega =\{x:V\left(x\right)\leq \bar{V}\}$ is fixed-time reachable and positively invariant. Therefore, the system trajectory is guaranteed to enter the set *Ω* within the upper time bound T, which is independent of the initial condition, and*

$$T\leq \frac{1}{\vartheta a_{1}\left(1-\rho_{1}\right)}+\frac{1}{\vartheta a_{2}\left(\rho_{2}-1\right)}$$

**Theorem** **1.** *For the emergency rescue quadrotor UAV system described by (14) under the conditions of Assumption 1, including the prescribed bounds on the ESC–motor tracking mismatch and the uncertain structural resonance, if the stage-dependent estimator gains satisfy the Hurwitz and common quadratic Lyapunov conditions specified above for $R_{1}^{\left(k\right)}$ and $R_{2}^{\left(k\right)}$ in every mission stage k∈{1,2,3}, then, under arbitrary initial estimation errors, within each fixed mission stage k∈{1,2,3}, the estimation errors ${\tilde{x}}_{2}$ and $\tilde{\Delta }$ can enter and remain within the corresponding small bounded neighborhood within a fixed time independent of the initial conditions. At the stage-transition instants, the bounded jumps of Δ(t) induce only bounded variations in the estimation errors, and the estimator can re-enter the corresponding small bounded neighborhood of the subsequent mission stage within a fixed time after each transition. Moreover, the recovery time after each stage transition is uniformly bounded by the fixed-time upper bound associated with the corresponding post-transition mission stage. The above conclusion concerns the practical fixed-time convergence and post-transition recovery of the estimation errors. Its implication for the compensated closed-loop attitude-tracking system is given separately after the proof under Assumption 2.*

**Proof.**  Consider an arbitrary fixed mission stage k∈{1,2,3} where σ(t)=k. In this interval, the estimator gains are constant, and the estimation error dynamics can be written as

(20) $$\left\{\begin{array}{cc} {\dot{\tilde{x}}}_{2} & =\tilde{\Delta }-\Lambda_{1}^{\left(k\right)}\Phi_{m_{1}}\;\left({\tilde{x}}_{2}\right)-M_{1}^{\left(k\right)}\Phi_{n_{1}}\;\left({\tilde{x}}_{2}\right)\\ \dot{\tilde{\Delta }} & =\dot{\Delta }-\Lambda_{2}^{\left(k\right)}\Phi_{m_{2}}\;\left({\tilde{x}}_{2}\right)-M_{2}^{\left(k\right)}\Phi_{n_{2}}\;\left({\tilde{x}}_{2}\right)-\Gamma^{\left(k\right)}\tanh\;\left(\frac{{\tilde{x}}_{2}}{\varepsilon_{e}}\right) \end{array}\right.$$

Define the complete estimation-error vector as

$$e\left(t\right):=\mathrm{col}\left({\tilde{x}}_{2}\left(t\right),\tilde{\Delta }\left(t\right)\right)$$

Within the *k*-th fixed mission stage, where σ(t)=k, the same error vector is denoted by

$$e_{k}\left(t\right):=e\left(t\right)$$

According to the common quadratic Lyapunov condition in (19), for each fixed mission stage k∈{1,2,3}, let $P_{k}=P_{k}^{T}≻0$ denote the common Lyapunov matrix associated with $R_{1}^{\left(k\right)}$ and $R_{2}^{\left(k\right)}$. Define the auxiliary quadratic function

$$U_{k}\left(e_{k}\right)=e_{k}^{T}P_{k}e_{k}.$$

Since $P_{k}$ is symmetric positive definite, $U_{k}$ satisfies

$$\lambda_{\min}\left(P_{k}\right)∥e_{k}{∥}^{2}\leq U_{k}\left(e_{k}\right)\leq \lambda_{\max}\left(P_{k}\right){∥e_{k}∥}^{2}$$

The auxiliary function $U_{k}$ is used to verify the stability of the low-power and high-power limiting error systems and to quantify the bounded error variation at the mission-stage transitions. A strict Lyapunov function $V_{k}$ associated with the bi-limit homogeneous error system will be introduced below to establish the fixed-time dissipation inequality.

To separate the nominal fixed-time convergence mechanism from the bounded time-varying terms, the estimation-error dynamics in the *k*-th fixed mission stage are rewritten as

(21) $${\dot{e}}_{k}=F_{k}\left(e_{k}\right)+r_{k}\left(e_{k},t\right)$$

where the nominal error vector field is defined as $F_{k}\left(e_{k}\right)=\mathrm{col}\left(\tilde{\Delta }-\Lambda_{1}^{\left(k\right)}\Phi_{m_{1}}\left({\tilde{x}}_{2}\right)-M_{1}^{\left(k\right)}\Phi_{n_{1}}\left({\tilde{x}}_{2}\right),-\Lambda_{2}^{\left(k\right)}\Phi_{m_{2}}\left({\tilde{x}}_{2}\right)-M_{2}^{\left(k\right)}\Phi_{n_{2}}\left({\tilde{x}}_{2}\right)\right)$, and the remaining bounded term is $r_{k}\left(e_{k},t\right)=\mathrm{col}\left(0_{3},\dot{\Delta }\left(t\right)-\Gamma^{\left(k\right)}\tanh\left({\tilde{x}}_{2}/\varepsilon_{e}\right)\right)$. Here, $r_{k}\left(e_{k},t\right)$ denotes the bounded perturbation term in the estimation-error dynamics and should not be confused with the compensated closed-loop residual disturbance $r_{\Delta }\left(t\right)=\Delta \left(t\right)-{\hat{\Delta }}_{c}\left(t\right)$ defined in (15).

The nominal vector field $F_{k}\left(e_{k}\right)$ is homogeneous in the bi-limit. Its limiting vector field near the origin is

$$F_{m,k}\left(e_{k}\right)=\mathrm{col}\left(\tilde{\Delta }-\Lambda_{1}^{\left(k\right)}\Phi_{m_{1}}\left({\tilde{x}}_{2}\right),-\Lambda_{2}^{\left(k\right)}\Phi_{m_{2}}\left({\tilde{x}}_{2}\right)\right)$$

whereas its limiting vector field at infinity is

$$F_{n,k}\left(e_{k}\right)=\mathrm{col}\left(\tilde{\Delta }-M_{1}^{\left(k\right)}\Phi_{n_{1}}\left({\tilde{x}}_{2}\right),-M_{2}^{\left(k\right)}\Phi_{n_{2}}\left({\tilde{x}}_{2}\right)\right)$$

Since $m_{2}=2m_{1}-1$, the origin-limit system $F_{m,k}$ is homogeneous with respect to the dilation weights $\left(1,m_{1}\right)$ and has the negative homogeneity degree

$$d_{m}=m_{1}-1<0$$

Similarly, since $n_{2}=2n_{1}-1$, the infinity-limit system $F_{n,k}$ is homogeneous with respect to the dilation weights $\left(1,n_{1}\right)$ and has the positive homogeneity degree

$$d_{n}=n_{1}-1>0$$

Under the common quadratic Lyapunov condition specified above, the origin-limit and infinity-limit systems are globally asymptotically stable. Together with $d_{m}=m_{1}-1<0$ and $d_{n}=n_{1}-1>0$, the bi-limit homogeneity result guarantees global fixed-time stability of the nominal error system. To make the strict Lyapunov function and the subsequent coefficients numerically reproducible, the following constructive converse-Lyapunov representation is employed.

Let $\phi_{k}\left(\tau ,e_{k}\right)$ denote the solution of the nominal system ${\dot{e}}_{k}=F_{k}\left(e_{k}\right)$ with initial condition $e_{k}$. Introduce the homogeneous norms associated with the origin and infinity dilations as

$$\nu_{m}\left(e_{k}\right)={\left(\sum_{i=1}^{3}{\left|{\tilde{x}}_{2i}\right|}^{4}+\sum_{i=1}^{3}{\left|{\tilde{\Delta }}_{i}\right|}^{4/m_{1}}\right)}^{1/4},$$

$$\nu_{n}\left(e_{k}\right)={\left(\sum_{i=1}^{3}{\left|{\tilde{x}}_{2i}\right|}^{4}+\sum_{i=1}^{3}{\left|{\tilde{\Delta }}_{i}\right|}^{4/n_{1}}\right)}^{1/4}$$

Select $\ell_{m}=2.5$ and $\ell_{n}=5.074997$, which give

$$\rho_{m}=1+\frac{d_{m}}{\ell_{m}}=0.9,\;\rho_{n}=1+\frac{d_{n}}{\ell_{n}}=1.088670$$

Let χ:[0,∞)→[0,1] be the continuously differentiable transition function

$$\chi \left(s\right)=\left\{\begin{array}{cc} 1, & 0\leq s\leq 1,\\ 1-3{\left(s-1\right)}^{2}+2{\left(s-1\right)}^{3}, & 1<s<2,\\ 0, & s\geq 2 \end{array}\right.$$

Define

$$q_{k}\left(e_{k}\right)=\chi \;\left({∥e_{k}∥}_{2}\right)\nu_{m}^{\ell_{m}+d_{m}}\left(e_{k}\right)+\left[1-\chi \;\left({∥e_{k}∥}_{2}\right)\right]\nu_{n}^{\ell_{n}+d_{n}}\left(e_{k}\right)$$

A strict Lyapunov function for the nominal bi-limit homogeneous error system is then constructed as

$$V_{0k}\left(e_{k}\right)=\int_{0}^{\infty }q_{k}\;\left(\phi_{k}\left(\tau ,e_{k}\right)\right)\;d\tau$$

Because the nominal error system is globally fixed-time stable, the above integral is finite for every $e_{k}$. To remove the scaling ambiguity, define

$$c_{V_{k}}=\min_{{∥e_{k}∥}_{2}=1}V_{0k}\left(e_{k}\right),\;V_{k}\left(e_{k}\right)=\frac{V_{0k}\left(e_{k}\right)}{c_{V_{k}}}$$

and

$${\tilde{q}}_{k}\left(e_{k}\right)=\frac{q_{k}\left(e_{k}\right)}{c_{V_{k}}}$$

The flow-integral identity gives

$${\left.D^{+}V_{k}\right|}_{F_{k}}=-{\tilde{q}}_{k}\left(e_{k}\right)$$

Define the stage-dependent nominal dissipation coefficient by

$$d_{k}^{⋆}:=\underset{e_{k}\neq 0}{\inf}\frac{{\tilde{q}}_{k}\left(e_{k}\right)}{V_{k}^{\rho_{m}}\left(e_{k}\right)+V_{k}^{\rho_{n}}\left(e_{k}\right)}$$

The matching bi-limit orders of ${\tilde{q}}_{k}$ and $V_{k}$, together with continuity on the intermediate compact annulus, ensure that $d_{k}^{⋆}>0$. Consequently, the nominal error dynamics satisfy

(22) $${\left.D^{+}V_{k}\right|}_{F_{k}}\leq -d_{k}^{⋆}V_{k}^{\rho_{m}}-d_{k}^{⋆}V_{k}^{\rho_{n}}$$

It remains to evaluate the influence of the bounded term $r_{k}\left(e_{k},t\right)$. According to Assumption 1, within the *k*-th fixed mission stage, the lumped fault disturbance satisfies

$${∥\dot{\Delta }\left(t\right)∥}_{2}\leq {\bar{\rho }}_{k}$$

Moreover, because the hyperbolic tangent function is evaluated componentwise,

$${∥\tanh\;\left(\frac{{\tilde{x}}_{2}}{\varepsilon_{e}}\right)∥}_{2}\leq \sqrt{3}$$

Therefore, the bounded term defined above satisfies

(23) $$∥r_{k}\left(e_{k},t\right)∥\leq {\bar{\rho }}_{k}+\sqrt{3}{∥\Gamma^{\left(k\right)}∥}_{2}=:{\bar{r}}_{k}$$

In the revised disturbance model, ${\bar{\rho }}_{k}$ includes the variation-rate contributions of the ESC–motor tracking mismatch, actuator faults, environmental disturbances, payload variations, near-ground aerodynamic effects, unmodeled dynamics, and the bounded structural-resonance component within the prescribed frequency range. The value of ${\bar{\rho }}_{k}$ is evaluated separately within each fixed mission stage, while the bounded variations at the two transition instants are treated through the jump condition of Theorem 1.

Since $r_{k}\left(e_{k},t\right)$ enters only the lumped fault-disturbance error channel, its contribution to the upper Dini derivative of $V_{k}$ is

$$\left|\nabla V_{k}^{T}r_{k}\right|\leq {\bar{r}}_{k}∥\frac{\partial V_{k}}{\partial \tilde{\Delta }}∥$$

To make the residual bound explicit, define the stage-dependent gradient envelope

$$\psi_{k}\left(s\right):=\underset{\left\{e_{k}:V_{k}\left(e_{k}\right)=s\right\}}{\sup}∥\frac{\partial V_{k}\left(e_{k}\right)}{\partial \tilde{\Delta }}∥,\;s\geq 0$$

with $\psi_{k}\left(0\right)=0$. Since $V_{k}$ is continuously differentiable, radially unbounded, and homogeneous in the bi-limit, $\psi_{k}\left(s\right)$ is finite for every s≥0 and has a lower growth order than the corresponding nominal dissipation terms in the origin and infinity limits.

To remove the arbitrariness in the absorption parameters, a common absorption ratio κ=0.1 is selected for all three mission stages. Define

$${\bar{c}}_{k}:=\underset{s\geq 0}{\sup}{\left[{\bar{r}}_{k}\psi_{k}\left(s\right)-\kappa d_{k}^{⋆}s^{\rho_{m}}-\kappa d_{k}^{⋆}s^{\rho_{n}}\right]}_{+},$$

where ${\left[z\right]}_{+}=\max\left\{z,0\right\}$. The bi-limit growth orders of the normalized strict Lyapunov function ensure that the above supremum is finite. By construction, the perturbation contribution satisfies

(24) $$\nabla V_{k}^{T}r_{k}\leq \kappa d_{k}^{⋆}V_{k}^{\rho_{m}}+\kappa d_{k}^{⋆}V_{k}^{\rho_{n}}+{\bar{c}}_{k}$$

Combining Equations (22) and (24) gives

$$D^{+}V_{k}\leq -\left(1-\kappa \right)d_{k}^{⋆}V_{k}^{\rho_{m}}-\left(1-\kappa \right)d_{k}^{⋆}V_{k}^{\rho_{n}}+{\bar{c}}_{k}$$

Accordingly, the stage-dependent coefficients are explicitly defined as

$${\bar{a}}_{1k}=\left(1-\kappa \right)d_{k}^{⋆},\;{\bar{a}}_{2k}=\left(1-\kappa \right)d_{k}^{⋆},\;{\bar{c}}_{k}=\underset{s\geq 0}{\sup}{\left[{\bar{r}}_{k}\psi_{k}\left(s\right)-\kappa d_{k}^{⋆}\left(s^{\rho_{m}}+s^{\rho_{n}}\right)\right]}_{+}$$

Hence, with κ=0.1,

$${\bar{a}}_{1k}={\bar{a}}_{2k}=0.9d_{k}^{⋆}$$

and the complete estimation-error dynamics satisfy

(25) $$D^{+}V_{k}\leq -{\bar{a}}_{1k}V_{k}^{\rho_{m}}-{\bar{a}}_{2k}V_{k}^{\rho_{n}}+{\bar{c}}_{k}$$

Choose an arbitrary constant $\vartheta_{k}\in \left(0,1\right)$. Since ${\bar{a}}_{1k}>0$, ${\bar{a}}_{2k}>0$, $0<\rho_{m}<1<\rho_{n}$, and the function

$$h_{k}\left(s\right)={\bar{a}}_{1k}s^{\rho_{m}}+{\bar{a}}_{2k}s^{\rho_{n}}$$

is continuous and strictly increasing for s>0, there exists a unique ${\bar{V}}_{k}>0$ satisfying

$${\bar{a}}_{1k}{\bar{V}}_{k}^{\rho_{m}}+{\bar{a}}_{2k}{\bar{V}}_{k}^{\rho_{n}}=\frac{{\bar{c}}_{k}}{1-\vartheta_{k}}$$

Define the stage-dependent residual set as

$$\Omega_{k}:=\left\{e_{k}:V_{k}\left(e_{k}\right)\leq {\bar{V}}_{k}\right\}$$

For any $e_{k}\notin \Omega_{k}$, one has $V_{k}>{\bar{V}}_{k}$, and therefore

$${\bar{c}}_{k}<\left(1-\vartheta_{k}\right)\left({\bar{a}}_{1k}V_{k}^{\rho_{m}}+{\bar{a}}_{2k}V_{k}^{\rho_{n}}\right)$$

Substituting this inequality into the preceding Lyapunov inequality gives

$$D^{+}V_{k}\leq -\vartheta_{k}{\bar{a}}_{1k}V_{k}^{\rho_{m}}-\vartheta_{k}{\bar{a}}_{2k}V_{k}^{\rho_{n}},\;e_{k}\notin \Omega_{k}$$

According to Lemma 1, the set $\Omega_{k}$ is fixed-time reachable and positively invariant. Moreover, the estimation-error vector $e_{k}$ enters $\Omega_{k}$ within the stage-dependent fixed-time upper bound

(26) $$T_{k}\leq \frac{1}{\vartheta_{k}{\bar{a}}_{1k}\left(1-\rho_{m}\right)}+\frac{1}{\vartheta_{k}{\bar{a}}_{2k}\left(\rho_{n}-1\right)}$$

The construction leading to (26) removes the scaling and parameter-selection ambiguity in the converse Lyapunov argument. For each mission stage, $V_{k}$ is obtained from the normalized flow-integral construction, $d_{k}^{⋆}$ is evaluated by the stated global minimization, and ${\bar{c}}_{k}$ is obtained from the corresponding one-dimensional supremum. Therefore, ${\bar{a}}_{1k}$, ${\bar{a}}_{2k}$, ${\bar{c}}_{k}$, $V_{k}$, and $T_{k}$ are reproducible stage-dependent quantities determined by the final estimator gains and disturbance-variation bounds while remaining independent of the initial estimation errors. Therefore, both ${\bar{V}}_{k}$ and $T_{k}$ are computable stage-dependent quantities and are independent of the initial estimation errors. Consequently, within the *k*-th fixed mission stage, the angular-velocity estimation error ${\tilde{x}}_{2}$ and the lumped fault-disturbance estimation error $\tilde{\Delta }$ achieve practical fixed-time convergence to the stage-dependent bounded neighborhood $\Omega_{k}$.

The above result guarantees practical fixed-time convergence to a stage-dependent bounded residual set rather than exact reconstruction of every high-frequency resonance component. The size of the residual set depends on ${\bar{\rho }}_{k}$, the estimator gains, the smoothing parameters, and the prescribed resonance-frequency range. In particular, the result does not provide a uniform estimation bound when the resonance frequency or excitation amplitude is allowed to increase without bound.

Consider a mission-stage transition at $t_{i}$ from stage $k^{-}$ to stage $k^{+}$. The lumped disturbance is allowed to undergo a bounded jump

$$J_{i}=\Delta \;\left(t_{i}^{+}\right)-\Delta \;\left(t_{i}^{-}\right),\;{∥J_{i}∥}_{2}\leq {\bar{J}}_{i}$$

At $t_{i}$, the stage-dependent disturbance composition, actuator-effectiveness parameters, and estimator gain set may be updated. However, the physical and dynamic states are not reset. In the absence of impulsive torque inputs, the attitude and angular-velocity states $x_{1}\left(t\right)$ and $x_{2}\left(t\right)$, the ESC–motor state $u_{a}\left(t\right)$, and the structural modal states $q_{r}\left(t\right)$ and ${\dot{q}}_{r}\left(t\right)$ introduced in Equation (8) remain continuous. The estimator states ${\hat{x}}_{2}\left(t\right)$ and $\hat{\Delta }\left(t\right)$, together with the internal states of the compensation filter $Q_{c}\left(s\right)$, are also carried continuously across the transition. Moreover, since the coefficients of $Q_{c}\left(s\right)$ are not switched, the filtered estimate and the compensation torque satisfy

$${\hat{\Delta }}_{c}\;\left(t_{i}^{+}\right)={\hat{\Delta }}_{c}\;\left(t_{i}^{-}\right),\;u_{\mathrm{comp}}\;\left(t_{i}^{+}\right)=u_{\mathrm{comp}}\;\left(t_{i}^{-}\right)$$

The continuity of these dynamic states does not require the algebraically constructed lumped disturbance Δ(t) to be continuous. A bounded update of the disturbance composition, actuator effectiveness, payload condition, or other stage-dependent parameters may produce the bounded jump $J_{i}$. Such an update may cause a finite change in the corresponding state derivatives, but it does not generate an impulsive state jump. Consequently,

$${\tilde{x}}_{2}\;\left(t_{i}^{+}\right)={\tilde{x}}_{2}\;\left(t_{i}^{-}\right)$$

and

$$\begin{array}{cc} \tilde{\Delta }\;\left(t_{i}^{+}\right) & =\Delta \;\left(t_{i}^{+}\right)-\hat{\Delta }\;\left(t_{i}^{+}\right)\\ & =\Delta \;\left(t_{i}^{-}\right)+J_{i}-\hat{\Delta }\;\left(t_{i}^{-}\right)\\ & =\tilde{\Delta }\;\left(t_{i}^{-}\right)+J_{i} \end{array}$$

Therefore,

$$e\;\left(t_{i}^{+}\right)=e\;\left(t_{i}^{-}\right)+E_{i},\;E_{i}=\mathrm{col}\left(0,J_{i}\right)$$

In addition, since $r_{\Delta }\left(t\right)=\Delta \left(t\right)-{\hat{\Delta }}_{c}\left(t\right)$, as defined in Equation (15), and ${\hat{\Delta }}_{c}\left(t\right)$ remains continuous,

$$r_{\Delta }\;\left(t_{i}^{+}\right)-r_{\Delta }\;\left(t_{i}^{-}\right)=J_{i}$$

and hence

$${∥r_{\Delta }\;\left(t_{i}^{+}\right)-r_{\Delta }\;\left(t_{i}^{-}\right)∥}_{2}\leq {\bar{J}}_{i}$$

Thus, the mission-stage transition produces at most bounded jumps in the disturbance-estimation error and the compensated residual disturbance, while the physical, estimator, and compensation-filter states remain continuous.

Hence, if the pre-transition estimation error is bounded, the post-transition auxiliary function $U_{k^{+}}\left(t_{i}^{+}\right)$ and the corresponding error state $e(t_{i}^{+})$ are also bounded. Therefore, the bounded disturbance jump changes only the initial error value of the subsequent mission stage and does not invalidate the stage-wise fixed-time convergence property.

Let $T_{\mathrm{rec},i}$ denote the recovery time after the *i*-th stage transition from the pre-transition stage $k^{-}$ to the post-transition stage $k^{+}$. More precisely, define

$$T_{\mathrm{rec},i}:=\inf\left\{\tau \geq 0:e\left(t\right)\in \Omega_{k^{+}},\;\forall \;t\geq t_{i}+\tau \;\mathrm{while}\;\sigma \left(t\right)=k^{+}\right\},\;i=1,2$$

As established above, the bounded disturbance jump at $t_{i}$ produces only a bounded post-transition error $e(t_{i}^{+})$. More importantly, the fixed-time upper bound $T_{k^{+}}$ obtained for the post-transition mission stage is independent of the initial estimation error. Therefore, applying the fixed-stage convergence result to the post-transition error state gives

$$T_{\mathrm{rec},i}\leq T_{k^{+}},\;i=1,2$$

A uniform recovery-time upper bound for the complete takeoff–mission execution–landing process can thus be defined as

(27) $$T_{\mathrm{rec}}^{\max}:=\max_{k\in \{1,2,3\}}T_{k}$$

Consequently,

$$T_{\mathrm{rec},i}\leq T_{k^{+}}\leq T_{\mathrm{rec}}^{\max},\;i=1,2$$

Hence, within each fixed mission stage, the angular-velocity estimation error ${\tilde{x}}_{2}$ and the lumped fault-disturbance estimation error $\tilde{\Delta }$ enter and remain in the corresponding stage-dependent bounded neighborhood $\Omega_{k}$ within a fixed time independent of the initial estimation errors. At each mission-stage transition, the bounded jump of Δ(t) induces only a bounded change in the estimation error. The estimator subsequently re-enters the bounded neighborhood associated with the new mission stage within the uniform recovery-time bound $T_{\mathrm{rec}}^{\max}$. Therefore, the proposed estimator achieves stage-wise practical fixed-time convergence and uniformly bounded post-transition recovery of the estimation errors throughout the complete takeoff–mission execution–landing process. This completes the proof. □

The stage-wise bound in (26) is a global initial-condition-independent upper bound constructed using the constant worst-case perturbation envelope ${\bar{c}}_{k}$. Because the maximum admissible disturbance-variation contribution is treated as a persistent input over the complete mission stage, the resulting bound is generally conservative and is intended to establish the practical fixed-time property rather than to predict the recovery time of a particular mission trajectory. Section 4.3 therefore evaluates the actual post-transition recovery behavior directly from the final closed-loop simulation data using a common causal short-time RMS criterion. These trajectory-dependent numerical results complement, but do not constitute a direct numerical evaluation of, the global analytical bound in (26).

**Corollary** **1** 
(Closed-loop boundedness under estimator-based compensation)**.** *Under the conditions of Theorem 1 and Assumption 2, suppose that the compensation filter $Q_{c}\left(s\right)$ is stable, has finite initial states, and is neither switched nor reset at mission-stage transitions. Then, within each fixed mission stage, the attitude-tracking error of the compensated closed-loop system is uniformly ultimately bounded. Moreover, a bounded jump in the lumped disturbance at a mission-stage transition produces only a bounded closed-loop transient.*

**Proof.**  Define the mismatch between the raw disturbance estimate and the filtered estimate as $e_{f}\left(t\right)=\hat{\Delta }\left(t\right)-{\hat{\Delta }}_{c}\left(t\right)$. According to the definition of the compensated residual disturbance in Equation (15), $r_{\Delta }\left(t\right)=\Delta \left(t\right)-{\hat{\Delta }}_{c}\left(t\right)$. Since the disturbance-estimation error is defined as $\tilde{\Delta }\left(t\right)=\Delta \left(t\right)-\hat{\Delta }\left(t\right)$, the compensated residual disturbance can be decomposed as

$$\begin{array}{cc} r_{\Delta }\left(t\right) & =\Delta \left(t\right)-\hat{\Delta }\left(t\right)+\hat{\Delta }\left(t\right)-{\hat{\Delta }}_{c}\left(t\right)\\ & =\tilde{\Delta }\left(t\right)+e_{f}\left(t\right) \end{array}$$

For a fixed mission stage *k*, Theorem 1 guarantees that the estimation-error vector enters the bounded residual set $\Omega_{k}$ within a fixed time. Define the corresponding disturbance-estimation error bound as ${\bar{\delta }}_{k}:=\sup_{e_{k}\in \Omega_{k}}{∥\tilde{\Delta }∥}_{2}$. Because $\Omega_{k}$ is bounded, it follows that ${\bar{\delta }}_{k}<\infty$.

Moreover, Δ(t) is bounded under Assumption 1. Consequently, $\hat{\Delta }\left(t\right)=\Delta \left(t\right)-\tilde{\Delta }\left(t\right)$ remains bounded after the estimation error enters $\Omega_{k}$. Since $Q_{c}\left(s\right)$ is stable and has finite internal states, there exists a finite constant ${\bar{\delta }}_{f,k}>0$ such that ${∥e_{f}\left(t\right)∥}_{2}\leq {\bar{\delta }}_{f,k}$.

Therefore, the compensated residual disturbance satisfies

$$\begin{array}{cc} {∥r_{\Delta }\left(t\right)∥}_{2} & \leq {∥\tilde{\Delta }\left(t\right)∥}_{2}+{∥e_{f}\left(t\right)∥}_{2}\\ & \leq {\bar{\delta }}_{k}+{\bar{\delta }}_{f,k}\\ & =:{\bar{\delta }}_{r,k} \end{array}$$

According to Assumption 2, the closed-loop Lyapunov function satisfies ${\dot{V}}_{c}\leq -\alpha_{c}{∥e_{c}∥}_{2}^{2}+\beta_{c}{∥e_{c}∥}_{2}{∥r_{\Delta }∥}_{2}$. Using the above residual-disturbance bound gives ${\dot{V}}_{c}\leq -\alpha_{c}{∥e_{c}∥}_{2}^{2}+\beta_{c}{\bar{\delta }}_{r,k}{∥e_{c}∥}_{2}$, which can be rewritten as ${\dot{V}}_{c}\leq -{∥e_{c}∥}_{2}\left(\alpha_{c}{∥e_{c}∥}_{2}-\beta_{c}{\bar{\delta }}_{r,k}\right)$. Hence, ${\dot{V}}_{c}<0$ whenever

$${∥e_{c}∥}_{2}>\frac{\beta_{c}}{\alpha_{c}}{\bar{\delta }}_{r,k}$$

It follows that the closed-loop tracking error is uniformly ultimately bounded within each fixed mission stage, and the size of its ultimate neighborhood is determined by the disturbance-estimation error bound ${\bar{\delta }}_{k}$, the compensation-filter mismatch bound ${\bar{\delta }}_{f,k}$, and the closed-loop constants $\alpha_{c}$ and $\beta_{c}$.

At a mission-stage transition $t_{i}$, the continuity analysis given above yields

$$r_{\Delta }\;\left(t_{i}^{+}\right)-r_{\Delta }\;\left(t_{i}^{-}\right)=J_{i}$$

and

$${∥r_{\Delta }\;\left(t_{i}^{+}\right)-r_{\Delta }\;\left(t_{i}^{-}\right)∥}_{2}\leq {\bar{J}}_{i}$$

Because the physical states, estimator states, and compensation-filter states are not reset, the closed-loop tracking error $e_{c}\left(t\right)$ remains continuous at $t_{i}$. The bounded jump in $r_{\Delta }\left(t\right)$ may cause a finite change in ${\dot{V}}_{c}$ and in the derivatives of the closed-loop states, but it does not generate an impulsive state jump. Therefore, each mission-stage transition produces only a bounded closed-loop transient and does not destroy the boundedness of the compensated system. This completes the proof. □

Corollary 1 establishes Lyapunov-based boundedness of the estimator-compensated closed-loop system.

## 4. Instance Verification

This section verifies the performance of the proposed estimator through instance simulations.

### 4.1. Closed-Loop Multi-Stage Emergency Rescue Scenario and Parameter Settings

To verify the estimation performance of the proposed fixed-time fault-disturbance estimator, a scenario-based simulation experiment is constructed under a practical emergency rescue mission. The considered task is motivated by a post-disaster rescue scenario in a mountainous area. In this scenario, a quadrotor UAV takes off from a temporary rescue point, carries a small emergency payload or communication relay module, flies over the disaster area to perform low-altitude search, communication support, or material delivery, and finally descends to a temporary recovery area. During this process, the UAV may suffer from actuator efficiency loss, actuator bias faults, environmental airflow disturbances, payload-induced torque variations, near-ground aerodynamic effects, and unmodeled dynamic uncertainties. These effects are uniformly mapped into the angular-velocity dynamic channel and regarded as the true lumped fault disturbance to be estimated. To evaluate both the estimation performance and the closed-loop applicability of the proposed estimator, the estimator is embedded in a nonlinear attitude-control loop including a baseline attitude controller, an estimate-based disturbance-compensation channel, ESC dynamics, actuator faults, structural resonances, and stage-dependent environmental disturbances. The baseline controller and the compensation structure are introduced only as a closed-loop validation environment and are not claimed as new controller contributions.

The whole emergency rescue process is divided into three mission stages: takeoff, mission execution, and landing. The total simulation time is set as $T_{\mathrm{end}}=12\;s$, and the integration step size is selected as dt=0.0005s. The two stage-transition instants are selected as $t_{1}=3.0\;s$ and $t_{2}=9.0\;s$. Accordingly, the mission-stage variable is defined as

$$\sigma \left(t\right)=\left\{\begin{array}{cc} 1, & 0\leq t<t_{1}\\ 2, & t_{1}\leq t<t_{2}\\ 3, & t\geq t_{2} \end{array}\right.$$

In (5), σ(t)=1, σ(t)=2, and σ(t)=3 denote the takeoff, mission execution, and landing stages, respectively.

The quadrotor inertia matrix used in the nonlinear plant, baseline controller, and fault-disturbance mapping is selected as $J=\mathrm{diag}\left\{0.0081,\;0.0081,\;0.0142\right\}\;\mathrm{kg}\;m^{2}$. The physical initial states are specified as $x_{1}\left(0\right)=\frac{\pi }{180}{\left[5,\;-4,\;6\right]}^{T}\;\mathrm{rad}$ and $x_{2}\left(0\right)={\left[0,\;0,\;0\right]}^{T}\;\mathrm{rad}/s$.

The same inertia matrix is used throughout the closed-loop simulation, and the torque-domain fault and disturbance components are mapped into the angular-acceleration channel exclusively through $J^{-1}$. Therefore, no additional empirical input-gain matrix is introduced.

To generate the commanded torque online under a reproducible closed-loop maneuver, the desired attitude trajectory is defined as $x_{1d}\left(t\right)={\left[\phi_{d}\left(t\right),\;\theta_{d}\left(t\right),\;\psi_{d}\left(t\right)\right]}^{T}$. Let $s=\left(t-t_{1}\right)/\left(t_{2}-t_{1}\right)$. The reference attitude is prescribed as

$$x_{1d}\left(t\right)=\left\{\begin{array}{cc} 0_{3}, & 0\leq t<t_{1},\\ \frac{\pi }{180}\sin^{2}\left(\pi s\right)\left[\begin{array}{c} 8\sin(2\pi s)\\ 6\sin(4\pi s)\\ 10\sin(2\pi s) \end{array}\right], & t_{1}\leq t<t_{2},\\ 0_{3}, & t_{2}\leq t\leq T_{\mathrm{end}} \end{array}\right.$$

During takeoff and landing, the desired attitude is maintained at the level-flight condition, whereas smooth roll, pitch, and yaw maneuvers are introduced during the mission execution stage. The envelope $\sin^{2}\left(\pi s\right)$ ensures that both $x_{1d}\left(t\right)$ and ${\dot{x}}_{1d}\left(t\right)$ connect continuously at $t=t_{1}$ and $t=t_{2}$, thereby avoiding artificial torque impulses at the mission-stage transitions. The required reference derivatives are calculated analytically rather than by numerical differentiation.

To provide an explicit input for the near-ground aerodynamic model, the flight-height trajectory is defined using the smooth interpolation function

$$S\left(\xi \right)=3\xi^{2}-2\xi^{3},\;0\leq \xi \leq 1$$

Accordingly, the height profile is specified as

$$h\left(t\right)=\left\{\begin{array}{cc} h_{0}+\left(h_{m}-h_{0}\right)S\left(\frac{t}{t_{1}}\right), & 0\leq t<t_{1}\\ h_{m}, & t_{1}\leq t<t_{2},\\ h_{m}-\left(h_{m}-h_{f}\right)S\left(\frac{t-t_{2}}{T_{\mathrm{end}}-t_{2}}\right), & t_{2}\leq t\leq T_{\mathrm{end}} \end{array}\right.$$

where

$$h_{0}=0.20\;m,\;h_{m}=3.00\;m,\;h_{f}=0.20\;m$$

Thus, the vehicle smoothly ascends from the initial near-ground height to the mission altitude during the takeoff stage, maintains a constant altitude during mission execution, and returns to the near-ground region during landing. The height-dependent activation coefficient used in the subsequent near-ground disturbance model is calculated as

$$\chi_{\mathrm{ge}}\left(t\right)=\frac{1}{2}\left[1+\tanh\left(\frac{h_{g}-h\left(t\right)}{T_{h}}\right)\right]$$

with

$$h_{g}=1.20\;m,\;T_{h}=0.25\;m$$

Consequently, the near-ground disturbance is strongly activated during the initial takeoff and final landing intervals and becomes negligible at the nominal mission altitude.

Directly feeding the complete disturbance estimate $\hat{\Delta }\left(t\right)$ back into the actuator channel may amplify measurement noise and excite the uncertain high-frequency structural modes. Therefore, a bandwidth-limited compensation channel is introduced between the estimator and the commanded torque. The filtered disturbance estimate ${\hat{\Delta }}_{c}\left(t\right)$ is generated by

$${\dot{\hat{\Delta }}}_{c}\left(t\right)=-\Omega_{c}{\hat{\Delta }}_{c}\left(t\right)+\Omega_{c}\hat{\Delta }\left(t\right)$$

where

$$\Omega_{c}=\mathrm{diag}\left\{\omega_{c,\phi },\;\omega_{c,\theta },\;\omega_{c,\psi }\right\}>0$$

is the bandwidth matrix of the compensation filter. Equivalently, its transfer matrix can be written as

$$Q_{c}\left(s\right)=\Omega_{c}{\left(sI+\Omega_{c}\right)}^{-1},\;{\hat{\Delta }}_{c}\left(s\right)=Q_{c}\left(s\right)\hat{\Delta }\left(s\right)$$

Since $\hat{\Delta }\left(t\right)$ is an angular-acceleration estimate, it is converted into the torque domain through the inertia matrix *J*. The estimate-based compensation torque is therefore defined as

$$u_{\mathrm{comp}}\left(t\right)=-J{\hat{\Delta }}_{c}\left(t\right)$$

The final commanded torque supplied to the ESC–actuator subsystem is

$$u_{c}\left(t\right)=u_{b}\left(t\right)+u_{\mathrm{comp}}\left(t\right)=u_{b}\left(t\right)-J{\hat{\Delta }}_{c}\left(t\right)$$

Accordingly, the angular-velocity dynamics can be rewritten as

$${\dot{x}}_{2}=J^{-1}\left(-x_{2}\times Jx_{2}\right)+J^{-1}u_{b}+\Delta_{\mathrm{rem}}\left(t\right)$$

where the remaining disturbance after compensation is

$$\Delta_{\mathrm{rem}}\left(t\right)=\Delta \left(t\right)-{\hat{\Delta }}_{c}\left(t\right)=\left[\Delta \left(t\right)-\hat{\Delta }\left(t\right)\right]+\left[\hat{\Delta }\left(t\right)-{\hat{\Delta }}_{c}\left(t\right)\right]$$

The first term represents the estimation error of the proposed estimator, whereas the second term represents the bandwidth-limitation error introduced by the compensation filter. This decomposition explicitly retains the estimation delay and compensation-filter phase lag in the closed-loop model. For the nominal simulations, the compensation-filter bandwidth is selected as

$$\Omega_{c}=\mathrm{diag}\left\{12,\;12,\;10\right\}\;\mathrm{rad}/s$$

which is higher than the dominant attitude-control bandwidth but substantially lower than the prescribed structural-resonance frequencies. The initial filter state is set as

$${\hat{\Delta }}_{c}\left(0\right)=0_{3}$$

No reset is applied at the mission-stage transitions; hence,

$${\hat{\Delta }}_{c}\left(t_{k}^{+}\right)={\hat{\Delta }}_{c}\left(t_{k}^{-}\right),\;k=1,2$$

The phase lag introduced by $Q_{c}\left(s\right)$ is explicitly included in the subsequent phase-margin, gain-margin, and delay-margin evaluations. In the theoretical plant model and the proposed estimator, the known control input is hereafter identified as

$$u\left(t\right)\equiv u_{c}\left(t\right)$$

The difference between the commanded torque $u_{c}\left(t\right)$ and the actual actuator output is subsequently incorporated into the actuator-induced disturbance $d_{\mathrm{act}}\left(t\right)$.

The commanded torque $u_{c}\left(t\right)$ is transmitted to the propulsion system through the ESC and motor dynamics. To account for the finite actuator-response bandwidth and torque saturation, the equivalent three-axis ESC–actuator dynamics are described by

$$T_{\mathrm{esc}}{\dot{u}}_{a}\left(t\right)+u_{a}\left(t\right)={\mathrm{sat}}_{\bar{u}}\;\left(u_{c}\left(t\right)\right)$$

where $u_{a}\left(t\right)={\left[u_{a,\phi }\left(t\right),\;u_{a,\theta }\left(t\right),\;u_{a,\psi }\left(t\right)\right]}^{T}$ denotes the equivalent actuator torque before the efficiency-loss and bias faults, and

$$T_{\mathrm{esc}}=\mathrm{diag}\left\{T_{\mathrm{esc},\phi },\;T_{\mathrm{esc},\theta },\;T_{\mathrm{esc},\psi }\right\}$$

is the ESC–actuator time-constant matrix. The componentwise saturation operator is defined as

$${\left[{\mathrm{sat}}_{\bar{u}}\left(v\right)\right]}_{i}=\mathrm{sgn}\left(v_{i}\right)\min\{|v_{i}\left|,\;{\bar{u}}_{i}\right\}\;i\in \left\{\phi ,\theta ,\psi \right\}$$

where ${\bar{u}}_{i}$ denotes the maximum admissible torque in the corresponding attitude channel. The nominal actuator parameters are selected as

$$T_{\mathrm{esc}}=\mathrm{diag}\left\{0.025,\;0.025,\;0.035\right\}\;s$$

and

$$\bar{u}={\left[0.12,\;0.12,\;0.08\right]}^{T}\;N\;m$$

The actuator-effectiveness matrix is defined as

$$E\left(t\right)=\mathrm{diag}\{\eta_{\phi }\left(t\right),\;\eta_{\theta }\left(t\right),\;\eta_{\psi }\left(t\right)\}$$

where

$$\eta_{i}\left(t\right)=1-\rho_{i}H_{f}\left(t-t_{f,i}\right)$$

and the smooth fault-activation function is

$$H_{f}\left(t-t_{f}\right)=\frac{1}{2}\left[1+\tanh\left(\frac{t-t_{f}}{T_{f}}\right)\right]$$

The effectiveness-loss profiles used in the nominal scenario are

$$E\left(t\right)=\mathrm{diag}\left\{1-0.25H_{f}\left(t-4.5\right),\;1-0.20H_{f}\left(t-6.0\right),\;1-0.15H_{f}\left(t-7.5\right)\right\}$$

In addition, the actuator-bias torque is specified as

$$b_{u}\left(t\right)=\left[\begin{array}{c} 0.0025H_{f}\left(t-5.5\right)\\ -0.0020H_{f}\left(t-6.8\right)\\ 0.0015H_{f}\left(t-8.0\right) \end{array}\right]\;N\;m$$

The actual torque delivered to the quadrotor rotational dynamics is then expressed as

$$\tau_{a}\left(t\right)=E\left(t\right)u_{a}\left(t\right)+b_{u}\left(t\right)$$

Relative to the known commanded torque $u_{c}\left(t\right)$, the actuator-induced mismatch torque is defined as

$$\tau_{\mathrm{act}}\left(t\right)=\tau_{a}\left(t\right)-u_{c}\left(t\right)=E\left(t\right)u_{a}\left(t\right)+b_{u}\left(t\right)-u_{c}\left(t\right)$$

Equivalently, it can be decomposed as

$$\tau_{\mathrm{act}}\left(t\right)=\left[E\left(t\right)-I\right]u_{c}\left(t\right)+E\left(t\right)\left[u_{a}\left(t\right)-u_{c}\left(t\right)\right]+b_{u}\left(t\right)$$

The three terms on the right-hand side, respectively, represent the effectiveness-loss contribution, the ESC tracking and saturation mismatch, and the additive actuator-bias torque. The corresponding equivalent angular-acceleration disturbance is obtained through the physical inertia matrix as

$$d_{\mathrm{act}}\left(t\right)=J^{-1}\tau_{\mathrm{act}}\left(t\right)=J^{-1}\left[E\left(t\right)u_{a}\left(t\right)+b_{u}\left(t\right)-u_{c}\left(t\right)\right]$$

Accordingly, the angular-velocity dynamics can be written as

$${\dot{x}}_{2}=J^{-1}\left(-x_{2}\times Jx_{2}\right)+J^{-1}u_{c}+d_{\mathrm{act}}+d_{\mathrm{res}}+d_{\mathrm{env}}+d_{\mathrm{load}}+d_{\mathrm{ge}}+d_{\mathrm{dyn}}$$

Substituting the definition of $d_{\mathrm{act}}$ into the above equation gives

$$\begin{array}{cc} {\dot{x}}_{2}\left(t\right)= & J^{-1}\;\left[-x_{2}\left(t\right)\times Jx_{2}\left(t\right)\right]+J^{-1}\;\left[E\left(t\right)u_{a}\left(t\right)+b_{u}\left(t\right)\right]\\ & +d_{\mathrm{res}}\left(t\right)+d_{\mathrm{env}}\left(t\right)+d_{\mathrm{load}}\left(t\right)+d_{\mathrm{ge}}\left(t\right)+d_{\mathrm{dyn}}\left(t\right) \end{array}$$

which confirms that the commanded torque is not counted twice. The ESC state is not reset at the mission-stage transitions and therefore satisfies

$$u_{a}\left(t_{k}^{+}\right)=u_{a}\left(t_{k}^{-}\right),\;k=1,2$$

To explicitly represent uncertain high-frequency structural vibrations and their influence on both the rotational dynamics and the onboard measurements, a three-axis second-order modal model is incorporated into the closed-loop simulation. Let $q_{r}\left(t\right)={\left[q_{r,\phi }\left(t\right),\;q_{r,\theta }\left(t\right),\;q_{r,\psi }\left(t\right)\right]}^{T}$ denote the normalized structural modal coordinates. The modal dynamics are described by ${\ddot{q}}_{r}\left(t\right)+2Z_{r}\Omega_{r}{\dot{q}}_{r}\left(t\right)+\Omega_{r}^{2}q_{r}\left(t\right)=B_{r}s_{r}\left(t\right)$, where $\Omega_{r}=2\pi \mathrm{diag}\left\{f_{r,\phi },\;f_{r,\theta },\;f_{r,\psi }\right\}$ is the natural-frequency matrix, $Z_{r}=\mathrm{diag}\left\{\zeta_{\phi },\;\zeta_{\theta },\;\zeta_{\psi }\right\}$ is the damping-ratio matrix, and $B_{r}$ is the modal input matrix. The normalized modal formulation $B_{r}=\Omega_{r}^{2}$ is adopted so that $q_{r}\left(t\right)$ and the excitation $s_{r}\left(t\right)$ have the same normalized dimension.

The excitation of the *i*-th structural mode is defined as

$$s_{r,i}\left(t\right)=\alpha_{r}\left(t\right)A_{r,i}\sin\left(2\pi f_{e,i}t+\varphi_{i}\right)+\beta_{r,i}u_{a,i}\left(t\right),\;i\in \left\{\phi ,\theta ,\psi \right\}$$

where $A_{r,i}$, $f_{e,i}$, and $\varphi_{i}$ denote the periodic-excitation amplitude, excitation frequency, and initial phase, respectively. The coefficient $\beta_{r,i}$ represents the coupling between the actuator output and the corresponding structural mode. To avoid an artificial discontinuity at the beginning of the simulation, the excitation envelope is selected as

$$\alpha_{r}\left(t\right)=1-\exp\left(-\frac{t}{T_{r,a}}\right)\;T_{r,a}=0.15\;s$$

The structural-resonance torque acting on the rotational dynamics is expressed as

$$\tau_{\mathrm{res}}\left(t\right)=C_{r,q}q_{r}\left(t\right)+C_{r,v}{\dot{q}}_{r}\left(t\right)$$

where $C_{r,q}$ and $C_{r,v}$ denote the modal displacement-to-torque and modal velocity-to-torque matrices, respectively. The corresponding equivalent angular-acceleration disturbance is

$$d_{\mathrm{res}}\left(t\right)=J^{-1}\tau_{\mathrm{res}}\left(t\right)$$

For the nominal multi-stage rescue scenario, the structural parameters are selected as

$$f_{r}={\left[35,\;55,\;80\right]}^{T}\;\mathrm{Hz}$$

$$\zeta ={\left[0.040,\;0.035,\;0.030\right]}^{T}$$

$$f_{e}={\left[34.5,\;54.0,\;78.5\right]}^{T}\;\mathrm{Hz}$$

$$A_{r}={\left[0.015,\;0.012,\;0.010\right]}^{T}$$

and

$$\varphi ={\left[0,\;\pi /3,\;\pi /2\right]}^{T}$$

The actuator-to-mode coupling coefficients are set as $\beta_{r}={\left[0.20,\;0.18,\;0.15\right]}^{T}\;{\left(N\;m\right)}^{-1}$. The modal output matrices are selected as $C_{r,q}=10^{-3}\mathrm{diag}\left\{1.2,\;1.0,\;0.9\right\}\;N\;m$ and $C_{r,v}=10^{-6}\mathrm{diag}\left\{4.0,\;3.5,\;3.0\right\}\;N\;m\;s$.

The initial structural states are specified as

$$q_{r}\left(0\right)=0_{3},\;{\dot{q}}_{r}\left(0\right)=0_{3}$$

The modal states are not reset at the mission-stage transitions and therefore satisfy

$$q_{r}\left(t_{k}^{+}\right)=q_{r}\left(t_{k}^{-}\right),\;{\dot{q}}_{r}\left(t_{k}^{+}\right)={\dot{q}}_{r}\left(t_{k}^{-}\right),\;k=1,2$$

To evaluate the estimator beyond a single nominal resonance realization, the uncertain modal parameters are subsequently varied within

$$f_{r,i}\in \left[20,100\right]\;\mathrm{Hz},$$

$$\zeta_{i}\in \left[0.01,0.10\right],$$

$$A_{r,i}\in \left[0.005,0.020\right],$$

and

$$\frac{\left|f_{e,i}-f_{r,i}\right|}{f_{r,i}}\leq 0.05$$

The frequency-sweep and randomized-parameter tests are conducted within these prescribed ranges. Therefore, the subsequent results address uncertain high-frequency structural resonances within the specified frequency interval rather than only a single deterministic vibration frequency.

In addition to actuator faults, the UAV is affected by stage-dependent rescue-environment disturbances. In the takeoff stage, near-ground aerodynamic effects, local airflow around the temporary takeoff point, and payload-induced attitude imbalance are considered. In the mission execution stage, abrupt gusts, thermal airflow, building-induced airflow, dust flow, and payload variation are introduced. In the landing stage, near-ground aerodynamic effects around the temporary recovery area and attitude-adjustment uncertainties are considered. These disturbances are described by the environmental disturbance $d_{\mathrm{env}}\left(t\right)$, the payload-induced disturbance $d_{\mathrm{load}}\left(t\right)$, the near-ground aerodynamic disturbance $d_{\mathrm{ge}}\left(t\right)$, and the unmodeled dynamic disturbance $d_{\mathrm{dyn}}\left(t\right)$.

In the present simulation scenario, $d_{\mathrm{ge}}\left(t\right)$ is constructed as a bounded phenomenological disturbance activated by the flight height during takeoff and landing. This low-order representation is intended to reproduce the stage-dependent activation and principal variation trend of the near-ground aerodynamic effect rather than the detailed rotor-wake and pressure-cushion dynamics. Higher-order effects associated with the vertical velocity, rotor-speed variations, vehicle attitude, ground-surface characteristics, and unsteady wake interactions are not separately synthesized in the current simulations. Their bounded equivalent influence within the considered modeling and measurement bandwidths can be represented through the unmodeled-dynamics channel $d_{\mathrm{dyn}}\left(t\right)$. The structural-resonance contribution is represented separately by $d_{\mathrm{res}}\left(t\right)$ and is therefore not absorbed into $d_{\mathrm{dyn}}\left(t\right)$ in the following simulations.

Based on the preceding actuator, structural-resonance, environmental, payload, near-ground, and unmodeled-dynamics descriptions, the true lumped fault disturbance acting on the angular-velocity channel is defined as

$$\Delta \left(t\right)=d_{\mathrm{act}}\left(t\right)+d_{\mathrm{res}}\left(t\right)+d_{\mathrm{env}}\left(t\right)+d_{\mathrm{load}}\left(t\right)+d_{\mathrm{ge}}\left(t\right)+d_{\mathrm{dyn}}\left(t\right)$$

All the terms in Δ(t) are expressed in the angular-acceleration domain and have the unit $\mathrm{rad}/s^{2}$. In particular, the actuator-induced and structural-resonance components are given by

$$d_{\mathrm{act}}\left(t\right)=J^{-1}\left[E\left(t\right)u_{a}\left(t\right)+b_{u}\left(t\right)-u_{c}\left(t\right)\right]$$

and

$$d_{\mathrm{res}}\left(t\right)=J^{-1}\left[C_{r,q}q_{r}\left(t\right)+C_{r,v}{\dot{q}}_{r}\left(t\right)\right]$$

The three-axis lumped disturbance is written as

$$\Delta \left(t\right)=\left[\begin{array}{c} \Delta_{\phi }\left(t\right)\\ \Delta_{\theta }\left(t\right)\\ \Delta_{\psi }\left(t\right) \end{array}\right]$$

Accordingly, the closed-loop angular-velocity dynamics are expressed as

$${\dot{x}}_{2}\left(t\right)=J^{-1}\left[-x_{2}\left(t\right)\times Jx_{2}\left(t\right)\right]+J^{-1}u_{c}\left(t\right)+\Delta \left(t\right)$$

Substituting the definition of $d_{\mathrm{act}}\left(t\right)$ into the above equation yields

$${\dot{x}}_{2}\left(t\right)=J^{-1}\left[-x_{2}\left(t\right)\times Jx_{2}\left(t\right)\right]+J^{-1}\left[E\left(t\right)u_{a}\left(t\right)+b_{u}\left(t\right)\right]+d_{\mathrm{res}}\left(t\right)+d_{\mathrm{env}}\left(t\right)+d_{\mathrm{load}}\left(t\right)+d_{\mathrm{ge}}\left(t\right)+d_{\mathrm{dyn}}\left(t\right)$$

This equivalent expression shows that the commanded torque $u_{c}\left(t\right)$ is not counted twice. The difference between the actual actuator output and the commanded torque is included only through $d_{\mathrm{act}}\left(t\right)$, whereas the structural-resonance torque is included only through $d_{\mathrm{res}}\left(t\right)$.

The measured angular-velocity contamination $C_{\omega r}{\dot{q}}_{r}\left(t\right)$ is not included in Δ(t) because it acts through the IMU measurement channel rather than through the physical rotational dynamics.

The true lumped disturbance is generated online together with the closed-loop plant dynamics. In particular, $d_{\mathrm{act}}\left(t\right)$ depends on the commanded torque $u_{c}\left(t\right)$, the ESC state $u_{a}\left(t\right)$, and the time-varying effectiveness matrix E(t), whereas $d_{\mathrm{res}}\left(t\right)$ is generated by the structural modal states. Therefore, the true disturbance is no longer independent of the closed-loop control trajectory.

After incorporating the baseline closed-loop controller, the bandwidth-limited disturbance-compensation channel, the ESC–actuator dynamics, the structural-resonance modes, and the vibration-contaminated angular-rate measurements, the estimator parameters are adjusted under the complete closed-loop simulation conditions. The power parameters are selected as $m_{1}=0.75$ and $n_{1}=1.45$, satisfying $1/2<m_{1}<1<n_{1}$. Accordingly, $m_{2}=2m_{1}-1=0.50$ and $n_{2}=2n_{1}-1=1.90$. The smoothing parameter of the hyperbolic-tangent injection term is set as $\varepsilon_{e}=0.01$.

The gain selection follows the Hurwitz and common quadratic Lyapunov conditions established in Section 3. Within the admissible gain region, the gains are adjusted by jointly considering the disturbance-estimation response, recovery after mission-stage transitions, and amplification of structural-vibration and measurement-noise components. The final stage-dependent gain matrices used in the closed-loop simulations are selected as follows.

For the takeoff stage, 0≤t<3.0s,

$$\begin{array}{cccccc} \Lambda_{1}^{\left(1\right)} & =27I_{3}, & M_{1}^{\left(1\right)} & =6I_{3}, & & \\ \Lambda_{2}^{\left(1\right)} & =648I_{3}, & M_{2}^{\left(1\right)} & =168I_{3}, & \Gamma^{\left(1\right)} & =96I_{3} \end{array}$$

For the mission execution stage, 3.0≤t<9.0s,

$$\begin{array}{cccccc} \Lambda_{1}^{\left(2\right)} & =25.5I_{3}, & M_{1}^{\left(2\right)} & =6I_{3}, & & \\ \Lambda_{2}^{\left(2\right)} & =612I_{3}, & M_{2}^{\left(2\right)} & =156I_{3}, & \Gamma^{\left(2\right)} & =96I_{3} \end{array}$$

For the landing stage, 9.0≤t≤12.0s,

$$\begin{array}{cccccc} \Lambda_{1}^{\left(3\right)} & =24I_{3}, & M_{1}^{\left(3\right)} & =5.25I_{3}, & & \\ \Lambda_{2}^{\left(3\right)} & =576I_{3}, & M_{2}^{\left(3\right)} & =168I_{3}, & \Gamma^{\left(3\right)} & =84I_{3} \end{array}$$

Here, $I_{3}$ denotes the 3×3 identity matrix, and $\Gamma^{\left(k\right)}$ denotes the smooth injection gain. These parameter sets are used directly in all subsequent closed-loop simulations without additional gain scaling.

The initial attitude and angular velocity of the quadrotor are specified as

$$x_{1}\left(0\right)=\frac{\pi }{180}{\left[\begin{array}{ccc} 5 & -4 & 6 \end{array}\right]}^{T},\;x_{2}\left(0\right)={\left[\begin{array}{ccc} 0 & 0 & 0 \end{array}\right]}^{T}$$

At the initial instant, the desired attitude and its first two derivatives satisfy

$$x_{1d}\left(0\right)=0_{3},\;{\dot{x}}_{1d}\left(0\right)=0_{3},\;{\ddot{x}}_{1d}\left(0\right)=0_{3}$$

The initial states of the proposed estimator and the bandwidth-limited disturbance-compensation filter are selected as

$${\hat{x}}_{2}\left(0\right)=0_{3},\;\hat{\Delta }\left(0\right)=0_{3},\;{\hat{\Delta }}_{c}\left(0\right)=0_{3}$$

The angular-rate filter used by the baseline controller is initialized as

$$x_{2,f}\left(0\right)=0_{3}$$

where $x_{2,f}$ denotes the filtered angular-rate signal employed only by the baseline controller. The proposed estimator continues to use the vibration-contaminated measured angular rate $x_{2,m}$.

The initial structural-resonance states are set to

$$q_{r}\left(0\right)=0_{3},\;{\dot{q}}_{r}\left(0\right)=0_{3}$$

To avoid an artificial ESC command-tracking mismatch at the beginning of the simulation, the actuator state is initialized using the saturated initial commanded torque,

$$u_{a}\left(0\right)={\mathrm{sat}}_{\bar{u}}\;\left(u_{c}\left(0\right)\right)={\mathrm{sat}}_{\bar{u}}\;\left(u_{b}\left(0\right)\right)$$

where

$$u_{b}\left(0\right)=x_{2}\left(0\right)\times Jx_{2}\left(0\right)+J\left[{\ddot{x}}_{1d}\left(0\right)-K_{p}\left(x_{1}\left(0\right)-x_{1d}\left(0\right)\right)-K_{d}\left(x_{2}\left(0\right)-{\dot{x}}_{1d}\left(0\right)\right)\right]$$

For the parameters employed in the simulation, this gives

$$u_{a}\left(0\right)=u_{b}\left(0\right)={\left[\begin{array}{ccc} -0.01272 & 0.01018 & -0.02082 \end{array}\right]}^{T}\;N\;m$$

which lies within the prescribed actuator limits. In addition, the initial mission stage and flight height are specified as

$$\sigma \left(0\right)=1,\;h\left(0\right)=h_{0}=0.20\;m$$

Since $x_{2}\left(0\right)={\hat{x}}_{2}\left(0\right)$, the initial angular-velocity estimation error is zero, whereas the initial disturbance-estimation error is generally nonzero. The structural-vibration contribution and the prescribed measurement noise are retained in the measured angular-rate signal from the beginning of the simulation.

To provide a conventional observer benchmark, a second-order linear extended-state observer (LESO) is implemented in parallel with the proposed estimator. Let

$${\tilde{x}}_{2,L}=x_{2,m}-{\hat{x}}_{2,L}$$

where $x_{2,m}$ is the vibration-contaminated measured angular velocity. The LESO is defined as

$$\begin{array}{cc} {\dot{\hat{x}}}_{2,L} & =J^{-1}\left[-x_{2,m}\times Jx_{2,m}\right]+J^{-1}u_{c}+{\hat{\Delta }}_{L}+L_{1,L}{\tilde{x}}_{2,L},\\ {\dot{\hat{\Delta }}}_{L} & =L_{2,L}{\tilde{x}}_{2,L} \end{array}$$

The LESO gain matrices are selected according to

$$L_{1,L}=2\Omega_{L},\;L_{2,L}=\Omega_{L}^{2}$$

with

$$\Omega_{L}=\mathrm{diag}\left\{7,8,6\right\}\;\mathrm{rad}/s$$

Consequently,

$$\begin{array}{cc} L_{1,L} & =\mathrm{diag}\{14,16,12\}\\ L_{2,L} & =\mathrm{diag}\{49,64,36\} \end{array}$$

The initial LESO states are specified as

$${\hat{x}}_{2,L}\left(0\right)=0_{3},\;{\hat{\Delta }}_{L}\left(0\right)=0_{3}$$

For the estimator-level comparison, the proposed estimator and the LESO are operated in parallel on the same closed-loop plant trajectory. Both estimators use the same measured angular-velocity signal $x_{2,m}$, commanded torque $u_{c}$, ESC–actuator dynamics, actuator-fault profiles, structural-resonance realization, environmental and payload disturbances, measurement-noise realization, mission-stage transition instants, sampling period, integration step, and numerical integration method.

Only the bandwidth-limited estimate ${\hat{\Delta }}_{c}$ generated from the proposed estimator is connected to the compensation channel in this parallel-observer comparison. The LESO output ${\hat{\Delta }}_{L}$ is recorded only as an estimation benchmark and does not alter the control command or the true disturbance trajectory. Separate closed-loop cases are subsequently employed when comparing the compensation performance of the proposed estimator and the LESO.

**Figure 2:**
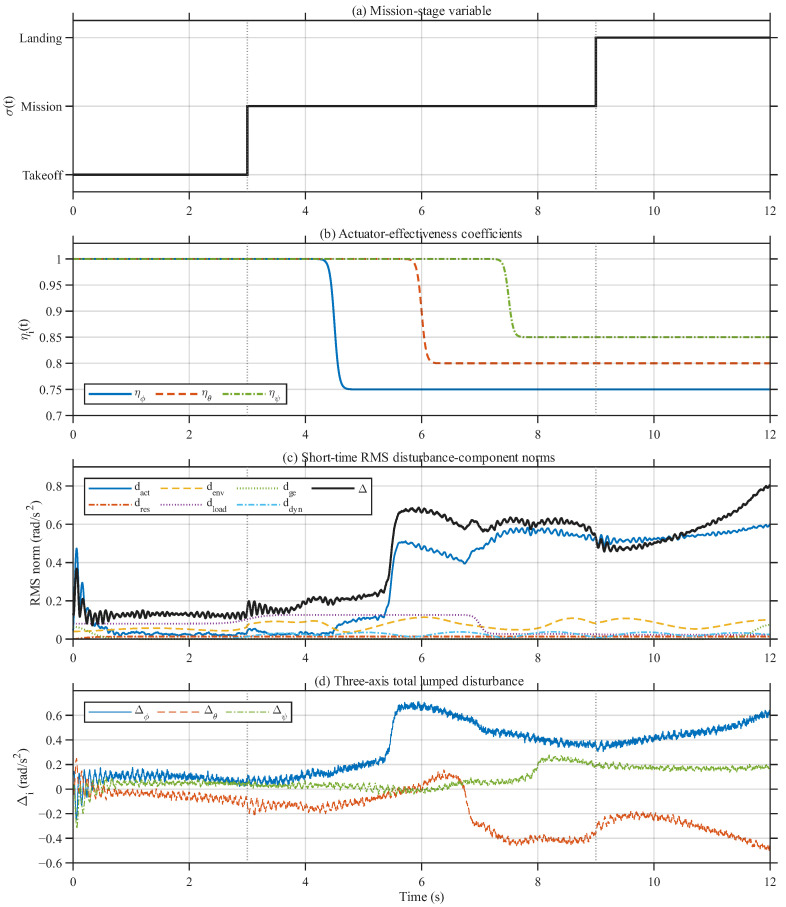
Multi-stage closed-loop fault-disturbance scenario.

Figure 2 presents the constructed multi-stage lumped fault-disturbance scenario, including the mission-stage variable, actuator effectiveness-loss faults, the norms of different disturbance components, and the three-axis components of the total lumped fault disturbance. This simulation scenario provides a controlled and repeatable multi-stage emergency rescue test case for evaluating the fixed-time estimation performance of the proposed estimator. The prescribed reference-attitude trajectory and bounded fault-disturbance activation profiles provide a controlled and repeatable closed-loop test case for evaluating the proposed estimator. They are intended to reproduce representative mission-stage variations rather than all the nonidealities encountered in an embedded flight system.

### 4.2. Closed-Loop Estimation Performance and Comparative Analysis

Based on the closed-loop emergency rescue scenario established in Section 4.1, this subsection evaluates the disturbance-estimation performance of the proposed fixed-time estimator under baseline attitude control, estimate-based compensation, ESC dynamics, actuator faults, structural resonance, and stage-dependent rescue-environment disturbances. The true lumped fault disturbance is generated online together with the closed-loop plant dynamics and is expressed as

$$\Delta \left(t\right)=d_{\mathrm{act}}\left(t\right)+d_{\mathrm{res}}\left(t\right)+d_{\mathrm{env}}\left(t\right)+d_{\mathrm{load}}\left(t\right)+d_{\mathrm{ge}}\left(t\right)+d_{\mathrm{dyn}}\left(t\right)$$

The raw output of the proposed estimator is denoted by $\hat{\Delta }\left(t\right)$, whereas the filtered signal actually used by the compensation channel is denoted by ${\hat{\Delta }}_{c}\left(t\right)$. Accordingly, the raw disturbance-estimation error and the residual disturbance acting on the compensated closed-loop system are, respectively, defined as

$$e_{\Delta }\left(t\right)=\Delta \left(t\right)-\hat{\Delta }\left(t\right),\;r_{\Delta }\left(t\right)=\Delta \left(t\right)-{\hat{\Delta }}_{c}\left(t\right)$$

**Figure 3:**
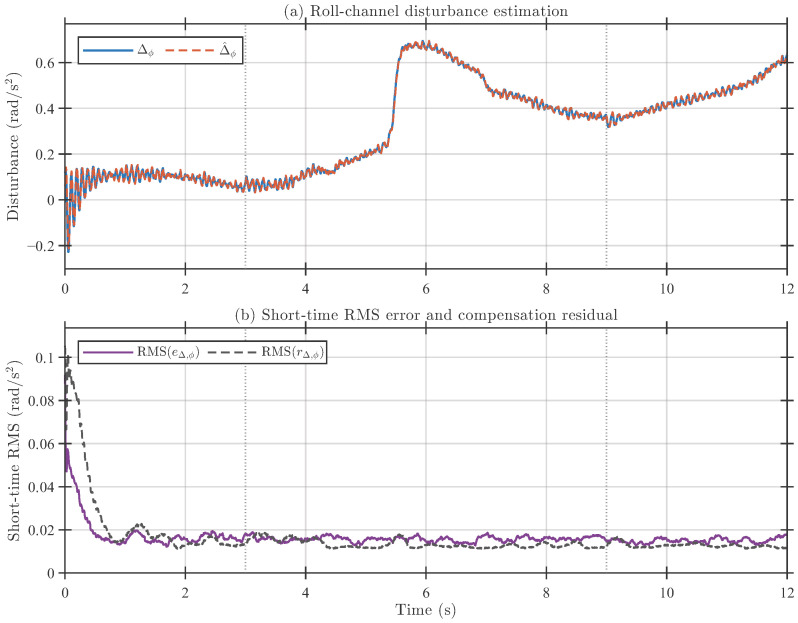
Roll-channel disturbance estimation and compensation residual.

**Figure 4:**
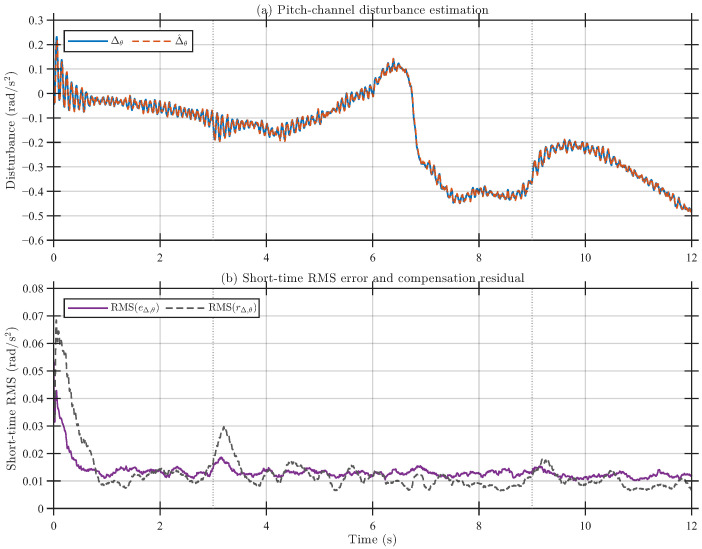
Pitch-channel disturbance estimation and compensation residual.

**Figure 5:**
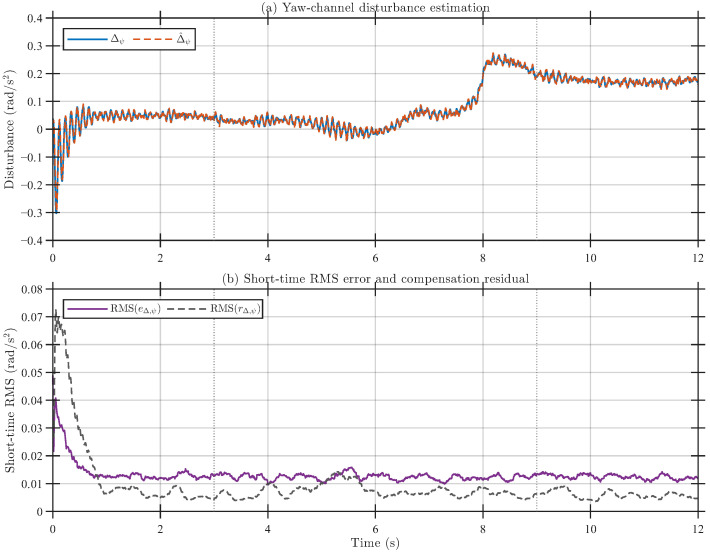
Yaw-channel disturbance estimation and compensation residual.

Figure 3, Figure 4 and Figure 5 present the roll-, pitch-, and yaw-channel results, respectively. To improve visual clarity, the upper subplot of each figure compares the true lumped disturbance with the raw estimator output after the same 20ms centered moving-average operation has been applied to both signals. The filtered compensation signal is omitted from the upper subplot because its close overlap with the other curves would obscure the direct comparison between the true disturbance and its estimate. The lower subplot presents the 0.20s trailing short-time RMS values of the raw estimation error and the compensation residual. The display operation does not enter the estimator, compensation channel, or closed-loop dynamics, and all the quantitative indices are calculated from the original unsmoothed causal signals.

Figure 3 presents the roll-channel lumped fault-disturbance estimation results under the closed-loop emergency rescue scenario. As shown in Figure 3a, the raw estimator output remains closely aligned with the true disturbance over the three mission stages, including the rapid variation around 5.3–5.7s. The remaining high-frequency fluctuations are reproduced without an observable macroscopic offset, while the maximum absolute stage-wise time differences obtained from the original unsmoothed signals are 4.0ms for the raw estimate and 7.0ms for the filtered compensation signal.

Figure 3b presents the 0.20s trailing short-time RMS values of the raw estimation error $e_{\Delta ,\phi }=\Delta_{\phi }-{\hat{\Delta }}_{\phi }$ and the compensation residual $r_{\Delta ,\phi }=\Delta_{\phi }-{\hat{\Delta }}_{c,\phi }$. The compensation residual is temporarily larger during initialization because of the compensation-filter transient. After this transient has decayed, both error envelopes remain bounded, and the compensation residual is generally smaller during the mission execution and landing stages. The overall raw-estimate RMSE and compensation-residual RMSE calculated from the unsmoothed signals are $0.016513\;\mathrm{rad}/s^{2}$ and $0.018237\;\mathrm{rad}/s^{2}$, respectively. The slightly larger overall residual RMSE is dominated by the initialization interval. During the mission execution and landing stages, the residual RMSE decreases from 0.015688 and $0.015170\;\mathrm{rad}/s^{2}$ to 0.013421 and $0.012785\;\mathrm{rad}/s^{2}$, corresponding to reductions of approximately 14.45% and 15.72%, respectively. Relative to the true-disturbance RMS of $0.372275\;\mathrm{rad}/s^{2}$, the compensation residual is attenuated by approximately 95.10%. These results show that the roll-channel compensation becomes effective after the initialization transient.

Figure 4 presents the pitch-channel disturbance-estimation result. As shown in Figure 4a, the pitch disturbance exhibits pronounced nonstationary variations over the three mission stages, together with persistent high-frequency components induced by structural vibration and environmental disturbances. Despite the changes in disturbance magnitude and variation trend, the raw estimator output remains closely aligned with the true disturbance during the rising, falling, and stage-transition intervals. No visually distinguishable macroscopic phase offset is observed.

Figure 4b shows the corresponding 0.20s trailing short-time RMS envelopes. Temporary increases occur during initialization, near the mission-stage transitions, and around the rapidly varying disturbance intervals, but both error envelopes remain bounded. The overall raw-estimate RMSE is $0.013443\;\mathrm{rad}/s^{2}$, whereas the overall compensation-residual RMSE is $0.014561\;\mathrm{rad}/s^{2}$. Relative to the true-disturbance RMS of $0.240328\;\mathrm{rad}/s^{2}$, the residual disturbance is attenuated by approximately 93.94%. The slightly larger overall residual RMSE is primarily caused by the compensation-filter initialization transient. During the mission execution and landing stages, the residual RMSE decreases from 0.013124 and $0.012032\;\mathrm{rad}/s^{2}$ to 0.012323 and $0.010351\;\mathrm{rad}/s^{2}$, corresponding to reductions of approximately 6.10% and 13.97%, respectively. Thus, after initialization, the filtered compensation signal effectively reduces the pitch-channel residual disturbance.

Figure 5 presents the closed-loop disturbance-estimation result for the yaw channel. Compared with the roll and pitch channels, the yaw disturbance has a smaller overall magnitude but contains a pronounced initialization transient and persistent high-frequency fluctuations. As shown in Figure 5a, the raw estimator output follows the dominant variation in the true disturbance during the initial rapid decay, the subsequent mission-stage variations, and the rapid increase around 8s. The two curves remain closely aligned over most of the steady-state intervals.

Figure 5b presents the 0.20s trailing short-time RMS values of the raw estimation error and compensation residual. The compensation-residual RMS is relatively large during initialization because of the combined effects of the compensation-filter initialization and the rapidly varying initial disturbance. After this transient has decayed, the compensation-residual RMS is generally smaller than the raw-estimation-error RMS, particularly during the mission execution and landing stages. The overall raw-estimate RMSE and compensation-residual RMSE are $0.013100\;\mathrm{rad}/s^{2}$ and $0.012216\;\mathrm{rad}/s^{2}$, respectively, representing a reduction of approximately 6.75%. During the mission execution and landing stages, the corresponding reductions are approximately 38.18% and 51.44%. Relative to the true-disturbance RMS of $0.118862\;\mathrm{rad}/s^{2}$, the compensation residual is attenuated by approximately 89.72%. These results demonstrate effective suppression of the dominant yaw-channel fault disturbance after the initialization transient.

Since the estimated disturbance is incorporated into the commanded torque through the filtered compensation signal ${\hat{\Delta }}_{c}\left(t\right)$, the time differences associated with both the raw estimator output and the actual compensation signal are evaluated. For channel i∈{ϕ,θ,ψ} and mission stage k∈{1,2,3}, the stage-wise time differences are defined as

$$\tau_{\hat{\Delta },i}^{\left(k\right)}=\underset{\tau \in [-\tau_{\max},\tau_{\max}]}{\arg\max}\;\mathrm{corr}\left(\Delta_{i}\left(t\right),{\hat{\Delta }}_{i}\left(t+\tau \right)\right),\;t\in I_{k}$$

and

$$\tau_{{\hat{\Delta }}_{c},i}^{\left(k\right)}=\underset{\tau \in [-\tau_{\max},\tau_{\max}]}{\arg\max}\;\mathrm{corr}\left(\Delta_{i}\left(t\right),{\hat{\Delta }}_{c,i}\left(t+\tau \right)\right),\;t\in I_{k}$$

where $I_{k}$ denotes the valid analysis interval of the *k*th mission stage. To prevent initialization transients, stage-transition variations, and high-frequency structural-resonance components from dominating the correlation results, the calculation is conducted separately within the three mission stages using equally processed 0.10 s dominant trends. The initial 0.50 s interval and the 0.20 s neighborhoods adjacent to the stage transitions are excluded. This identical trend processing is used only for time-difference diagnosis and does not alter, shift, or reconstruct the original causal estimation and compensation signals.

The maximum absolute stage-wise time differences of the raw estimates are 4.0, 4.5, and 1.5ms for the roll, pitch, and yaw channels, respectively, whereas the corresponding values of the filtered compensation signals are 7.0, 6.5, and 8.0ms. Therefore, the three-channel estimates remain closely synchronized with the true lumped fault disturbances, and no visually distinguishable macroscopic phase offset is observed in Figure 3, Figure 4 and Figure 5. These values are calculated from the original unsmoothed causal signals and are not reduced through curve shifting. The identical 20ms centered moving-average operation used in the upper subplots is introduced solely to improve graphical readability and does not enter the time-difference calculation, quantitative error evaluation, or closed-loop dynamics.

To distinguish the estimator accuracy from the disturbance attenuation achieved by the compensation channel, three error signals are introduced. The raw estimation error of the proposed estimator, the residual disturbance after filtered compensation, and the LESO estimation error are, respectively, defined as

$$e_{\Delta }\left(t\right)=\Delta \left(t\right)-\hat{\Delta }\left(t\right),\;r_{\Delta }\left(t\right)=\Delta \left(t\right)-{\hat{\Delta }}_{c}\left(t\right),\;e_{\Delta ,L}\left(t\right)=\Delta \left(t\right)-{\hat{\Delta }}_{L}\left(t\right)$$

where $\hat{\Delta }\left(t\right)$ denotes the raw output of the proposed estimator, ${\hat{\Delta }}_{c}\left(t\right)$ is the filtered compensation signal incorporated into the commanded torque, and ${\hat{\Delta }}_{L}\left(t\right)$ is the output of the parallel LESO. Accordingly, $e_{\Delta }\left(t\right)$ characterizes the estimation accuracy of the proposed estimator itself, whereas $r_{\Delta }\left(t\right)$ represents the disturbance remaining in the compensated closed-loop angular-velocity dynamics. The LESO error $e_{\Delta ,L}\left(t\right)$ is evaluated using the same true disturbance and closed-loop trajectory as those used for the proposed estimator.

The corresponding three-axis Euclidean error norms are given by

$${∥e_{\Delta }\left(t\right)∥}_{2}=\sqrt{e_{\Delta ,\phi }^{2}\left(t\right)+e_{\Delta ,\theta }^{2}\left(t\right)+e_{\Delta ,\psi }^{2}\left(t\right)},$$

$${∥r_{\Delta }\left(t\right)∥}_{2}=\sqrt{r_{\Delta ,\phi }^{2}\left(t\right)+r_{\Delta ,\theta }^{2}\left(t\right)+r_{\Delta ,\psi }^{2}\left(t\right)}$$

and

$${∥e_{\Delta ,L}\left(t\right)∥}_{2}=\sqrt{e_{\Delta ,L,\phi }^{2}\left(t\right)+e_{\Delta ,L,\theta }^{2}\left(t\right)+e_{\Delta ,L,\psi }^{2}\left(t\right)}$$

To provide a readable representation of the channel-wise estimation errors, Figure 3, Figure 4 and Figure 5 use the 0.20s trailing short-time RMS values of the raw estimation error and compensation residual. For a sampled error signal $q(t_{j})$, the short-time RMS is calculated as

$${\mathrm{RMS}}_{T_{w}}\left(q;t_{j}\right)=\sqrt{\frac{1}{N_{w,j}}\sum_{\ell \in W_{j}}{∥q(t_{\ell })∥}_{2}^{2}},\;T_{w}=0.20\;s$$

where

$$W_{j}=\left\{\ell :\max\left(0,t_{j}-T_{w}\right)\leq t_{\ell }\leq t_{j}\right\}$$

denotes the trailing sample window ending at $t_{j}$, and $N_{w,j}$ is the number of samples contained in that window. The same window length is applied to the raw estimation error and compensation residual in all three channels. This short-time RMS operation is used only for graphical presentation and does not alter the estimator output, compensation signal, overall RMSE values, or closed-loop plant dynamics. The 50ms short-time RMS window used subsequently in Figure 3, Figure 4 and Figure 5 is retained for estimator comparison and stage-transition recovery analysis.

**Figure 6:**
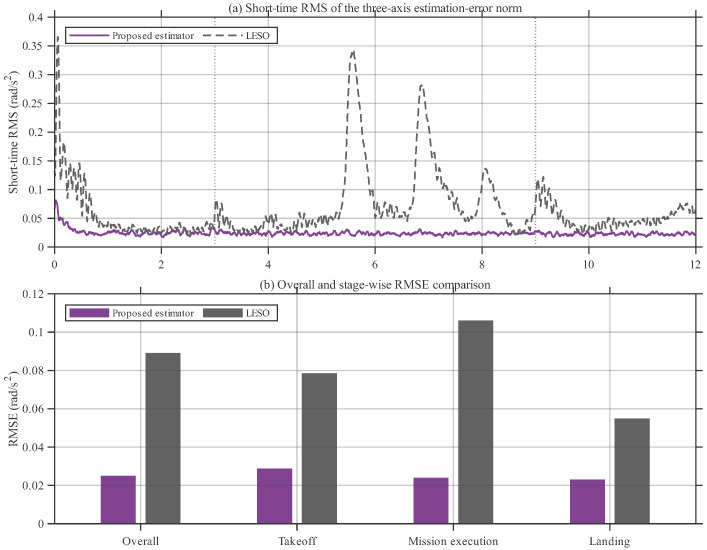
Comparison of disturbance-estimation error norms.

For the quantitative comparison in Figure 6, the overall vector RMSE of an error signal q(t) is defined as

$$\mathrm{RMSE}\left(q\right)=\sqrt{\frac{1}{N}\sum_{j=1}^{N}{∥q(t_{j})∥}_{2}^{2}}$$

while the RMSE over the *k*th mission stage is calculated as

$${\mathrm{RMSE}}^{\left(k\right)}\left(q\right)=\sqrt{\frac{1}{N_{k}}\sum_{t_{j}\in I_{k}}{∥q(t_{j})∥}_{2}^{2}}$$

where *N* and $N_{k}$ denote the numbers of samples over the complete mission and the *k*th mission stage, respectively. These definitions are applied consistently to the proposed estimator and the LESO.

For an estimator-level comparison, the proposed estimator and the LESO are operated in parallel using the same closed-loop angular-rate measurements, commanded torque, ESC dynamics, actuator faults, structural-resonance responses, environmental disturbances, integration step, and observer sampling period. The LESO bandwidth vector is selected as ${\left[7,8,6\right]}^{T}\;\mathrm{rad}/s$, following the bandwidth–noise tradeoff described in Section 4.1. Only the proposed estimator is connected to the compensation channel when generating the common closed-loop trajectory, whereas the LESO is evaluated offline as a parallel observer using exactly the same stored data. Therefore, the comparison isolates the disturbance-estimation performance and is not affected by differences between independently generated plant trajectories.

Figure 6a compares the 50 ms short-time RMS values of the three-axis estimation-error norms of the proposed estimator and the LESO. Both methods exhibit initialization transients, but the error of the proposed estimator decreases rapidly and subsequently remains within a relatively small neighborhood. By contrast, the LESO presents a larger initial error and several pronounced error peaks during the mission execution stage, particularly near the rapid actuator-fault and bias-fault variations. The proposed estimator maintains a comparatively smooth error response during these intervals, indicating improved adaptation to the stage-dependent and rapidly varying lumped fault disturbances.

Figure 6b reports the overall and stage-wise vector RMS values. The overall RMSE values of the proposed estimator and the LESO are approximately $0.025002\;\mathrm{rad}/s^{2}$ and $0.089050\;\mathrm{rad}/s^{2}$, respectively, corresponding to a reduction of approximately 71.92%. During the takeoff, mission execution, and landing stages, the proposed estimator yields RMSE values of approximately 0.028736, 0.023940, and $0.022988\;\mathrm{rad}/s^{2}$, whereas the corresponding LESO values are approximately 0.078539, 0.106144, and $0.054947\;\mathrm{rad}/s^{2}$. These results correspond to stage-wise reductions of approximately 63.31%, 77.45%, and 58.16%, respectively.

The largest improvement is obtained during the mission execution stage, in which actuator-effectiveness losses, actuator-bias faults, payload variation, structural resonance, and environmental disturbances act simultaneously. The results demonstrate that the proposed estimator provides lower estimation errors and faster recovery from rapidly varying fault-disturbance conditions than the LESO under the same closed-loop trajectory.

The results in Figure 3, Figure 4 and Figure 5 demonstrate that the proposed estimator can track the dominant variations in the lumped fault disturbances in all three attitude channels under the closed-loop emergency rescue scenario involving ESC dynamics, actuator faults, structural resonance, payload variation, near-ground effects, and stage-dependent environmental disturbances. The raw estimator outputs and filtered compensation signals remain closely aligned with the true disturbances, without visually distinguishable phase offsets. The maximum absolute stage-wise time differences of the raw estimates are 4.0, 4.5, and 1.5ms in the roll, pitch, and yaw channels, respectively, whereas those of the filtered compensation signals are 7.0, 6.5, and 8.0ms. Both the raw estimation errors and compensation residuals remain bounded throughout the complete mission. Although temporary increases occur during initialization and rapidly varying transition intervals, the compensation residuals are generally reduced after the transient response has decayed, particularly during the mission execution and landing stages.

The comparison in Figure 6 further shows that the proposed estimator provides consistently lower three-axis estimation errors than the LESO under the same closed-loop trajectory. The overall vector RMSE is reduced from approximately $0.089050\;\mathrm{rad}/s^{2}$ for the LESO to approximately $0.025002\;\mathrm{rad}/s^{2}$ for the proposed estimator, corresponding to an improvement of approximately 71.92%. The proposed estimator also achieves lower RMSE values during the takeoff, mission execution, and landing stages, with the largest improvement occurring during the mission execution stage, where multiple actuator faults, payload variation, structural resonance, and environmental disturbances act simultaneously. These results verify the estimation accuracy, transient-recovery capability, and practical applicability of the proposed estimator in the considered multi-stage closed-loop scenario. The recovery behavior following mission-stage transitions is examined further in Section 4.3, while robustness under stage-detection delays, parameter perturbations, measurement noise, inertia uncertainty, and resonance variations is evaluated in Section 4.4.

### 4.3. Stage-Transition Recovery Analysis

**Figure 7:**
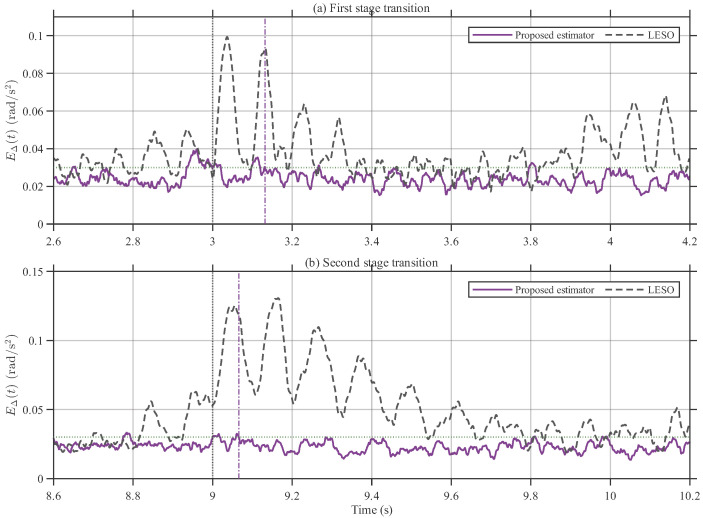
Post-transition estimation-error recovery.

To quantify the post-transition recovery performance under the resonance-related error fluctuations, Figure 7 presents local views of the causal short-time RMS envelopes of the three-axis disturbance-estimation error norms. A window length of $T_{w}=0.02\;s$ is employed. Considering the nonzero error floor caused by structural-resonance components and discrete-time implementation, the practical recovery threshold is selected as $\varepsilon_{\mathrm{rec}}=0.03\;\mathrm{rad}/s^{2}$. The recovery time is defined as the elapsed time from the corresponding transition instant until the short-time RMS error enters this accuracy band and remains within it for at least $T_{d}=0.10\;s$. The post-transition peak error is evaluated within the first 0.30s after each transition. Identical evaluation settings are applied to both estimators.

As shown in Figure 7a, at the first stage transition $t_{1}=3.0\;s$, the peak short-time RMS errors of the proposed estimator and the LESO are $0.035361\;\mathrm{rad}/s^{2}$ and $0.099331\;\mathrm{rad}/s^{2}$, respectively. Thus, the proposed estimator reduces the transition-induced peak error by 64.40%. Under the recovery threshold of $0.03\;\mathrm{rad}/s^{2}$, the proposed estimator recovers within 0.132s, whereas the LESO requires 5.593s to satisfy the same criterion. The corresponding reduction in recovery time is 97.64%.

At the second stage transition $t_{2}=9.0\;s$, as shown in Figure 7b, the difference between the two estimators becomes more pronounced. The proposed estimator limits the transition-induced error variation to a relatively small neighborhood, whereas the LESO produces a larger peak followed by an extended oscillatory decay. The quantitative peak and recovery-time results are summarized in Table 1.

As summarized in Table 1, the proposed estimator reduces the full-mission vector RMSE by 71.92%, with stage-wise reductions ranging from 58.16% to 77.45%. It also produces substantially smaller short-time RMS error peaks at both mission-stage transitions. After the first transition, the recovery time is reduced from 5.593s to 0.132s. After the second transition, the proposed estimator recovers within 0.066s, whereas the LESO does not return to the prescribed accuracy band before the end of the simulation. These results demonstrate the faster post-transition attenuation and stronger disturbance-estimation accuracy of the proposed estimator under the same closed-loop mission conditions.

Because the lumped fault disturbance remains time-varying and contains bounded resonance-related components within each mission stage, the estimation error is not expected to converge exactly to zero. Instead, the proposed estimator drives the error into a small bounded neighborhood after the initial transient and each stage transition. The practical recovery threshold used in Figure 7 is therefore consistent with the practical fixed-time convergence result established in Theorem 1.

The recovery times reported in Table 1 are obtained directly from the final closed-loop estimator trajectories using the causal short-time RMS criterion defined above. They are therefore mission-dependent numerical measures of the actual post-transition attenuation rather than evaluations of the global analytical upper bound in (26). Theorem 1 establishes the initial-condition-independent practical fixed-time property using global worst-case coefficients, whereas Figure 7 and Table 1 demonstrate the recovery performance realized under the prescribed takeoff–mission execution–landing trajectory. The analytical and numerical results thus address complementary aspects of the estimator: the global convergence guarantee and the mission-specific transient performance, respectively.

### 4.4. Implementation Sensitivity and Finite-Band Closed-Loop Validation

**Figure 8:**
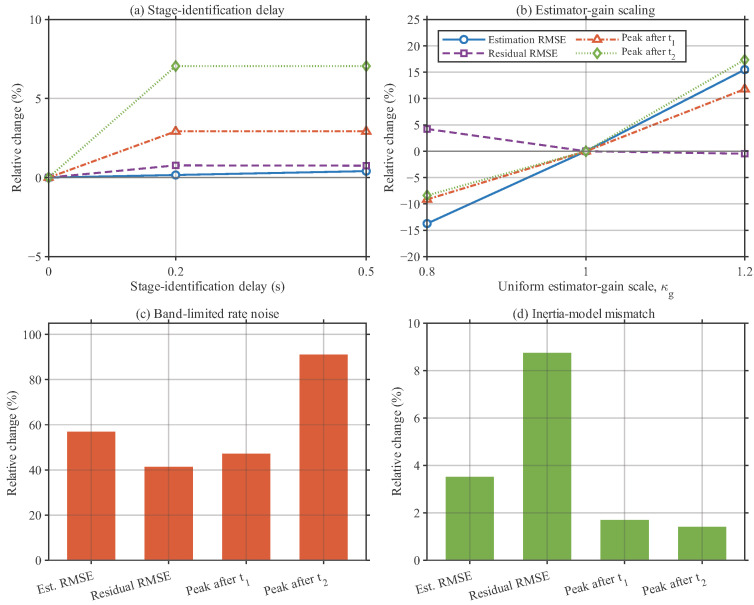
Implementation sensitivity of the proposed estimator.

**Figure 9:**
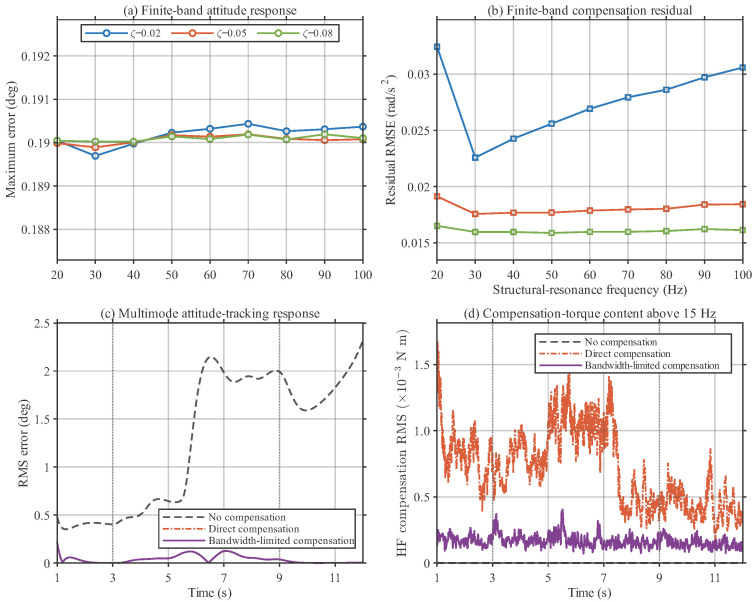
Finite-band resonance robustness and compensation comparison.

To evaluate the implementation sensitivity of the proposed estimator and the finite-band robustness of the associated compensation channel, additional closed-loop simulations are conducted under mission-stage identification delays, estimator-gain scaling, band-limited angular-rate measurement noise, rotational-inertia mismatch, and uncertain structural-resonance parameters. Figure 8 focuses on estimator-side implementation sensitivity, whereas Figure 9 examines the closed-loop response over the prescribed resonance band and compares no estimate compensation, direct estimate compensation, and bandwidth-limited estimate compensation. Unless otherwise stated, all cases use the baseline controller, compensation filter, ESC dynamics, actuator faults, environmental disturbances, initial conditions, integration step, and sampling period adopted in Figure 2.

First, the sensitivity to mission-stage identification delay is examined by replacing the actual stage signal supplied to the estimator with the delayed signal

$$\sigma_{d}\left(t\right)=\left\{\begin{array}{cc} 1, & 0\leq t<t_{1}+\tau_{\sigma }\\ 2, & t_{1}+\tau_{\sigma }\leq t<t_{2}+\tau_{\sigma }\\ 3, & t\geq t_{2}+\tau_{\sigma } \end{array}\right.$$

where $\tau_{\sigma }\in \left\{0,0.20,0.50\right\}\;s$. The case $\tau_{\sigma }=0$ corresponds to nominal stage identification, whereas $\tau_{\sigma }=0.20\;s$ and 0.50s represent moderate and relatively pronounced identification delays, respectively. In all three cases, the actual mission stage still changes at $t_{1}$ and $t_{2}$, and the physical plant, actuator faults, environmental and load disturbances, ESC dynamics, baseline controller, and compensation channel continue to evolve according to the actual stage signal σ(t). Only the stage signal used for estimator-gain scheduling is delayed. Consequently, during the intervals $\left[t_{1},t_{1}+\tau_{\sigma }\right)$ and $\left[t_{2},t_{2}+\tau_{\sigma }\right)$, the estimator temporarily retains the gain matrices associated with the preceding mission stage. This setting isolates the influence of delayed stage identification from changes in the actual plant and disturbance dynamics.

Second, the sensitivity to estimator-gain variation is investigated by uniformly scaling the stage-dependent gain matrices according to

$$G^{\left(k\right)}\left(\kappa_{g}\right)=\kappa_{g}G_{0}^{\left(k\right)}$$

where

$$G_{0}^{\left(k\right)}=\left\{\Lambda_{1}^{\left(k\right)},M_{1}^{\left(k\right)},\Lambda_{2}^{\left(k\right)},M_{2}^{\left(k\right)},\Gamma^{\left(k\right)}\right\},\;\kappa_{g}\in \left\{0.8,1.0,1.2\right\}$$

Here, $\kappa_{g}=1.0$ denotes the nominal tuning, whereas $\kappa_{g}=0.8$ and 1.2 represent uniformly reduced and increased estimator gains, respectively. For each tested value of $\kappa_{g}$ and each mission stage *k*, the scaled gains are substituted back into the stage-dependent matrices $R_{1}^{\left(k\right)}$ and $R_{2}^{\left(k\right)}$, and the corresponding Lyapunov equations are resolved. The dissipation coefficients ${\bar{a}}_{1k}$ and ${\bar{a}}_{2k}$, together with the residual bound ${\bar{c}}_{k}$, are then recalculated rather than inherited from the nominal case. The numerical verification confirms that $R_{1}^{\left(k\right)}$ and $R_{2}^{\left(k\right)}$ remain Hurwitz and that the recalculated dissipation coefficients remain positive for all three mission stages under each tested gain scale. Therefore, the cases shown in Figure 8b belong to a numerically verified discrete gain set. This verification applies only to $\kappa_{g}\in \left\{0.8,1.0,1.2\right\}$ and does not imply that the entire interval [0.8,1.2], or arbitrary gain scaling, is theoretically admissible.

Third, the influence of band-limited angular-rate measurement noise is evaluated by defining the measured angular-rate signal as

$$x_{2,m}\left(t\right)=x_{2}\left(t\right)+C_{\omega r}{\dot{q}}_{r}\left(t\right)+n_{\omega ,b}\left(t\right)$$

where $C_{\omega r}{\dot{q}}_{r}\left(t\right)$ denotes the deterministic angular-rate component induced by the structural vibration modes, and $n_{\omega ,b}\left(t\right)$ denotes the additional band-limited stochastic measurement noise. The latter is generated according to

$$n_{\omega ,b}\left(t\right)=H_{\omega }\left(s\right)n_{\omega ,0}\left(t\right)$$

where $H_{\omega }\left(s\right)$ is the adopted sensor-bandwidth model and $n_{\omega ,0}\left(t\right)$ is zero-mean random angular-rate noise with axis-dependent raw standard deviations

$$\mathrm{std}\;\left(n_{\omega ,0}\right)={\left[0.00150,\;0.00150,\;0.00120\right]}^{T}\;\mathrm{rad}/s$$

After passing through the sensor-bandwidth model, the resulting effective RMS levels are approximately

$$\mathrm{RMS}\;\left(n_{\omega ,b}\right)={\left[0.00017,\;0.00017,\;0.00013\right]}^{T}\;\mathrm{rad}/s$$

The filter $H_{\omega }\left(s\right)$ represents the finite bandwidth of the angular-rate measurement channel and is not introduced as an additional estimator correction or denoising mechanism. No additional attitude-angle measurement noise is imposed in this test. Figure 8c specifically evaluates the sensitivity of the proposed estimator to band-limited angular-rate measurement noise.

For the inertia-mismatch case, the actual plant inertia is selected as

$$J_{p}=\mathrm{diag}\left\{0.92J_{x},\;0.92J_{y},\;1.12J_{z}\right\}$$

while the estimator and the baseline controller retain the nominal inertia matrix *J*. The resulting state-dependent mismatch is therefore not explicitly compensated and enters the angular-rate dynamics as an additional modeling uncertainty.

To provide a consistent quantitative comparison among the implementation-sensitivity cases, the raw disturbance-estimation error and the compensation residual are defined as

$$e_{\Delta }\left(t\right)=\Delta \left(t\right)-\hat{\Delta }\left(t\right)$$

and

$$r_{\Delta }\left(t\right)=\Delta \left(t\right)-{\hat{\Delta }}_{c}\left(t\right)$$

respectively, where $\hat{\Delta }\left(t\right)$ is the direct output of the proposed estimator and ${\hat{\Delta }}_{c}\left(t\right)$ is the bandwidth-limited disturbance estimate actually supplied to the compensation branch. Accordingly, $e_{\Delta }\left(t\right)$ evaluates the intrinsic estimation accuracy, whereas $r_{\Delta }\left(t\right)$ represents the portion of the lumped disturbance remaining after implementable estimate compensation. Their full-horizon RMS measures are calculated as

$${\mathrm{RMSE}}_{e_{\Delta }}=\sqrt{\frac{1}{T_{\mathrm{end}}}\int_{0}^{T_{\mathrm{end}}}{∥e_{\Delta }\left(t\right)∥}_{2}^{2}\;dt}$$

and

$${\mathrm{RMSE}}_{r_{\Delta }}=\sqrt{\frac{1}{T_{\mathrm{end}}}\int_{0}^{T_{\mathrm{end}}}{∥r_{\Delta }\left(t\right)∥}_{2}^{2}\;dt}$$

To evaluate the local transient response after each mission-stage transition, a causal moving-RMS measure of the estimation error is further defined as

$$E_{\Delta ,\mathrm{rms}}\left(t\right)=\sqrt{\frac{1}{T_{w}}\int_{t-T_{w}}^{t}{∥e_{\Delta }\left(\tau \right)∥}_{2}^{2}\;d\tau },\;T_{w}=0.020\;s$$

For each transition, the recovery search begins at the detected post-transition peak. Practical recovery is declared when $E_{\Delta ,\mathrm{rms}}\left(t\right)$ becomes no greater than

$$\varepsilon_{\mathrm{rec}}=0.030\;\mathrm{rad}/s^{2}$$

and remains within this band continuously for at least 0.10s. The reported recovery time is the elapsed time from the detected post-transition peak to the first instant satisfying this persistence requirement. A zero recovery time indicates that the detected peak already lies inside the prescribed recovery band. The notation N/R indicates that the error does not re-enter and remain within this band before the end of the corresponding mission stage. This recovery criterion is an operational measure for comparing the simulated cases and should not be interpreted as a replacement for the theoretical fixed-time upper bound established in Theorem 1.

The relative effects of the four implementation factors are summarized in Figure 8. Figure 8a–d correspond to the mission-stage identification delay, uniform estimator-gain scaling, band-limited angular-rate measurement noise, and rotational-inertia mismatch, respectively. Each panel presents the percentage changes in the estimation RMSE, compensation-residual RMSE, and post-transition peak errors at $t_{1}$ and $t_{2}$ relative to the nominal case. A positive percentage indicates an increase in the corresponding error metric, whereas a negative percentage indicates a decrease. The corresponding absolute values and practical recovery times are reported separately in Table 2 because the zero and N/R recovery outcomes cannot be meaningfully represented as relative percentages.

Figure 8a shows that the considered stage-identification delays have only a minor influence on the overall estimation RMSE and compensation-residual RMSE. For the 0.50s delay, the estimation RMSE increases by 0.41%, while the post-transition peaks at $t_{1}$ and $t_{2}$ increase by 2.92% and 7.05%, respectively. The corresponding recovery times increase from 0.132s and 0.066s to 0.1605s and 0.1005s. Thus, the principal influence of the delayed stage signal appears in the local post-transition response rather than in the overall estimation accuracy.

Figure 8b confirms that the influence of uniform gain scaling is not monotonic. Reducing the gain scale to 0.8 decreases the overall estimation RMSE and transition-induced peaks but increases the compensation-residual RMSE by 4.22%. Increasing the gain scale to 1.2 slightly decreases the residual RMSE but increases the overall estimation RMSE by 15.48% and substantially prolongs the post-transition recovery. These results illustrate the tradeoff between rapid nonlinear correction, high-frequency estimation activity, and compensation accuracy.

Figure 8c indicates that the band-limited angular-rate measurement noise is the most influential implementation factor considered in this study. Relative to the nominal case, the estimation RMSE and compensation-residual RMSE increase by 56.96% and 41.33%, while the post-transition peaks increase by 47.17% and 91.00%. Although the estimation error remains finite throughout the simulation, it does not re-enter the prescribed nominal recovery band within the corresponding mission stages. Accordingly, no finite recovery time is assigned to this case.

Figure 8d shows that the considered inertia mismatch causes a 3.51% increase in the estimation RMSE and an 8.75% increase in the compensation-residual RMSE. The transition-induced peaks increase by only 1.70% and 1.41%, respectively. The recovery time after $t_{1}$ increases from 0.132s to 0.159s, whereas the recovery time after $t_{2}$ remains 0.066s. These results indicate moderate sensitivity to the prescribed inertia mismatch.

N/R indicates that the prescribed practical recovery band was not re-entered and continuously maintained for 0.10s before the end of the corresponding mission stage. It does not indicate divergence of the closed-loop response. A zero recovery time indicates that the detected post-transition peak already lies inside the prescribed recovery band.

To further evaluate the closed-loop behavior of the estimator-based compensation channel in the presence of uncertain structural resonance, a finite-band parametric sweep is conducted using the structural-mode model introduced in Section 2.2. The resonance frequency and damping ratio are selected as $f_{r}=20,30,\ldots ,100\;\mathrm{Hz}$ and ζ∈{0.02,0.05,0.08}, respectively. Combining the nine resonance frequencies with the three damping ratios produces 27 frequency–damping cases. The bandwidth-limited disturbance estimate is used in the compensation branch throughout this sweep. All the cases employ the same baseline controller, estimator parameters, compensation filter, ESC dynamics, actuator faults, environmental disturbances, initial conditions, integration step, sampling period, and random seed as the nominal closed-loop case. The maximum attitude-tracking error, compensation-residual RMSE, maximum commanded torque, and actuator-saturation count are evaluated over t≥1s, thereby excluding the initial-condition-dominated transient from the finite-band comparison.

In addition to the parametric sweep, a representative multimode structural-resonance case is introduced to compare different estimate-compensation implementations. The modal frequencies and damping ratios are selected as

$$f_{r}={\left[45,\;70,\;95\right]}^{T}\;\mathrm{Hz}$$

and

$$\zeta ={\left[0.02,\;0.03,\;0.02\right]}^{T}$$

respectively. The same 25% resonance-excitation enhancement and the same random seed are applied to all the compensation cases. Three implementations are considered: no estimate compensation, direct estimate compensation, and bandwidth-limited estimate compensation. For a unified representation, the compensation torque is defined as

$$u_{\mathrm{comp}}=-J{\hat{\Delta }}_{\mathrm{used}}$$

where

$${\hat{\Delta }}_{\mathrm{used}}=\left\{\begin{array}{cc} 0, & \mathrm{no}\;\mathrm{estimate}\;\mathrm{compensation},\\ \hat{\Delta }, & \mathrm{direct}\;\mathrm{estimate}\;\mathrm{compensation},\\ {\hat{\Delta }}_{c}, & \mathrm{bandwidth}\text{-}\mathrm{limited}\;\mathrm{estimate}\;\mathrm{compensation} \end{array}\right.$$

Here, $\hat{\Delta }$ is the direct estimator output, whereas ${\hat{\Delta }}_{c}$ is the estimate processed by the adopted compensation-bandwidth filter. This comparison changes only the implementation of the estimate-compensation branch; the baseline controller and all the other closed-loop settings remain unchanged. The high-frequency compensation-torque metric shown in Figure 9d is calculated from the spectral content of $u_{\mathrm{comp}}$ above 15Hz and should not be interpreted as the high-frequency content of the total commanded control torque.

Figure 9a,b summarize the results of the 27 finite-band resonance cases. The maximum attitude-tracking error remains between 0.18969° and 0.19043°, corresponding to a variation of less than 0.4% over the prescribed frequency and damping ranges. The compensation-residual RMSE remains between 0.015877 and $0.032435\;\mathrm{rad}/s^{2}$. Lower damping ratios generally produce larger residual errors, whereas no resonance frequency within the prescribed 20–100Hz band causes a pronounced increase in the attitude response. The maximum commanded torque remains between 0.00675 and 0.00691Nm, and no actuator saturation occurs in any of the 27 cases.

Figure 9c,d compare the three compensation implementations in the representative multimode case. Relative to the uncompensated case, bandwidth-limited compensation reduces the attitude RMSE by 96.36%, the maximum attitude error by 91.83%, and the compensation-residual RMSE by 94.07%. Compared with direct estimate compensation, the bandwidth-limited implementation produces a nearly identical attitude RMSE, with a difference of only 0.61%, while reducing the high-frequency compensation-torque RMSE by 77.79% and the high-frequency total-torque RMSE by 76.14%. The maximum commanded torque is also reduced by 5.73%. Therefore, the bandwidth-limited implementation retains comparable tracking performance to direct compensation while substantially decreasing the high-frequency content injected through the estimate-compensation branch.

Figure 8 and Figure 9 provide complementary numerical evidence regarding implementation sensitivity and finite-band closed-loop behavior. The considered stage-identification delays and inertia mismatch cause only moderate degradation, whereas the band-limited angular-rate measurement noise produces the most noticeable increase in the estimation and transition-peak errors. Over the prescribed 20–100Hz structural-resonance band, the attitude response and compensation residual remain bounded without actuator saturation.

Although the stage-identification-delay and band-limited measurement-noise tests introduced in Section 4.4 incorporate selected implementation nonidealities, the present results remain numerical and do not reproduce the complete sensor, processor, communication, actuator, and structural dynamics of an embedded UAV system. A feasible next-stage validation platform can be established using a processor-in-the-loop or hardware-in-the-loop architecture, in which a real-time six-degree-of-freedom quadrotor model is executed on a real-time simulation target and the proposed estimator is implemented on an actual flight-control processor at the intended sampling rate. The IMU interface can be configured to introduce measurement noise, bias, quantization, sampling jitter, transmission delay, and data loss, while an independent fault-injection module can reproduce actuator efficiency loss, actuator bias, ESC response lag and saturation, delayed or incorrect mission-stage signals, payload changes, and bounded aerodynamic disturbances. In addition to the estimation RMSE, transition-induced peak error, recovery time, and estimation lag, the validation should evaluate the worst-case execution time, missed sampling deadlines, processor and memory utilization, and numerical saturation events. After processor-in-the-loop and hardware-in-the-loop validation, tethered indoor tests with safely limited actuator degradation and controlled payload changes can be conducted before free-flight experiments.

## 5. Conclusions

This paper investigated the fixed-time fault-disturbance estimation problem for an emergency rescue quadrotor UAV operating throughout the takeoff–mission execution–landing process. A multi-stage lumped fault-disturbance model was established by incorporating actuator efficiency loss, actuator bias, ESC command-tracking mismatch, bounded structural-resonance effects within a prescribed frequency range, rescue-environment airflow disturbances, payload-induced disturbances, near-ground aerodynamic effects, and unmodeled dynamic uncertainties into the angular-velocity dynamic channel. Based on this model, a fixed-time fault-disturbance estimator with stage-dependent gains was developed and embedded in a closed-loop attitude-control system through a bandwidth-limited disturbance-compensation channel. The matrix-Lyapunov analysis established that the angular-velocity estimation error and the lumped fault-disturbance estimation error enter and remain in bounded neighborhoods within a fixed time independent of their initial conditions. Bounded disturbance variations at the mission-stage transitions were also shown to induce only bounded estimation transients, followed by fixed-time recovery. Moreover, the closed-loop analysis showed that bounded compensation residuals lead to uniformly ultimately bounded attitude-tracking errors. The baseline controller and compensation filter were introduced as a closed-loop validation environment and are not claimed as new controller contributions.

The closed-loop simulations under the multi-stage emergency rescue scenario demonstrated that the proposed estimator reconstructs the dominant variations in the roll-, pitch-, and yaw-channel lumped disturbances while maintaining bounded estimation errors after the two mission-stage transitions. Under the same closed-loop trajectory, the comparison with the LESO showed that the proposed estimator provides improved overall estimation accuracy and faster post-transition error attenuation according to the recalculated quantitative indices. The implementation-sensitivity tests further showed bounded estimation, compensation-residual, and attitude-tracking responses under the considered mission-stage identification delays, admissible estimator-gain variations, measurement noise, and rotational-inertia mismatch. In addition, the structural-resonance and compensation comparisons indicated that the bandwidth-limited compensation structure provides a suitable compromise between disturbance rejection and suppression of high-frequency control activity within the prescribed resonance range. Nevertheless, the current results remain simulation-based and employ low-order equivalent models for the ESC, structural modes, and near-ground aerodynamics. Future work will therefore implement the estimator and compensation algorithm on an actual flight-control processor and conduct processor-in-the-loop, hardware-in-the-loop, tethered-flight, and free-flight tests. Further investigation will also address observer–controller co-design, multimode structural dynamics, more detailed aerodynamic effects, sampling and communication delays, and actuator constraints.

## Figures and Tables

**Table 1:** Quantitative comparison of the proposed estimator and the LESO.

Performance Index	Proposed Estimator	LESO	Reduction Relative to LESO
Overall vector RMSE ($\mathrm{rad}/s^{2}$)	0.025002	0.089050	71.92%
Takeoff-stage RMSE ($\mathrm{rad}/s^{2}$)	0.028736	0.078539	63.41%
Mission-execution-stage RMSE ($\mathrm{rad}/s^{2}$)	0.023940	0.106144	77.45%
Landing-stage RMSE ($\mathrm{rad}/s^{2}$)	0.022988	0.054947	58.16%
Peak short-time RMS error after $t_{1}$ ($\mathrm{rad}/s^{2}$)	0.035361	0.099331	64.40%
Recovery time after $t_{1}$ (s)	0.132	5.593	97.64%
Peak short-time RMS error after $t_{2}$ ($\mathrm{rad}/s^{2}$)	0.032502	0.130666	75.13%
Recovery time after $t_{2}$ (s)	0.066	Not reached by $T_{\mathrm{end}}$	-

**Table 2:** Estimation and recovery metrics under different implementation-sensitivity conditions.

	Estimation	Residual		Recovery After		Recovery After
Test Condition	RMSE	RMSE	Peak at $t_{1}$	$t_{1}$ (s)	Peak at $t_{2}$	$t_{2}$ (s)
Nominal case	0.025002	0.026348	0.035361	0.1320	0.032502	0.0660
Stage delay: 0.20s	0.025042	0.026551	0.036395	0.1605	0.034794	0.1005
Stage delay: 0.50s	0.025103	0.026546	0.036395	0.1605	0.034794	0.1005
Gain scale: 0.8	0.021569	0.027460	0.032125	0.1160	0.029776	0
Gain scale: 1.2	0.028872	0.026222	0.039527	2.1030	0.038141	0.4385
Measurement noise	0.039243	0.037236	0.052039	N/R	0.062079	N/R
Inertia mismatch	0.025880	0.028653	0.035963	0.1590	0.032962	0.0660

## Data Availability

The data presented in this study are available on request from the corresponding author.
